# Assessment of Single‐Cycle M‐Protein Mutated Vesicular Stomatitis Virus as a Safe and Immunogenic Mucosal Vaccine Platform for SARS‐CoV‐2 Immunogen Delivery

**DOI:** 10.1002/advs.202404197

**Published:** 2024-11-11

**Authors:** En Zhang, Yong Ke, Weihong Ran, Yu Zhang, Ruihang Li, Xinkui Fang, Lei Wang, Baohong Zhang, Tao Sun

**Affiliations:** ^1^ School of Agriculture and Biology Shanghai Jiao Tong University Shanghai 200240 China; ^2^ Shanghai Municipal Veterinary Key Laboratory Shanghai 200240 China; ^3^ Engineering Research Center of Cell and Therapeutic Antibody, Ministry of Education, School of Pharmacy Shanghai Jiao Tong University Dongchuan Road, Minhang District Shanghai 200240 China

**Keywords:** COVID‐19, mucosal immunity, replication defective, Spike protein, vesicular stomatitis virus

## Abstract

The goal of the next‐generation COVID‐19 vaccine is to provide rapid respiratory tract protection with a single dose. Circulating antibodies do not protect the olfactory mucosa from viral infection, necessitating localized mucosal immunization. Live attenuated vesicular stomatitis virus (VSV_MT_)‐based COVID‐19 vaccines effectively stimulate mucosal immunity in animals, though safety concerns remain, particularly in immunocompromised populations. A viral vector capable of single‐cycle replication may face less stringent regulatory requirements. A replication‐defective VSV_MT_ is developed with its G protein replaced by a SARS‐CoV‐2 spike protein (S) mutant, where residues K986 and V987 are substituted by prolines (S2P). This studies show that single‐cycle VSV_MT_ encoding Omicron subvariant S2P (VSV_MT_‐S2P) is safe in both healthy and immunocompromised animals treated with cyclophosphamide (CP). Significant antibody and T‐cell responses against the spike protein are observed in VSV_MT_‐S2P vaccinated healthy animals. Intramuscular VSV_MT_‐S2P administration induces neutralizing antibody responses comparable to those from replication‐competent VSV_MT_‐S. In immunocompromised animals, lower and delayed immune responses are observed. Thus, single‐cycle M‐protein mutated VSV offers a safe and effective platform for SARS‐CoV‐2 immunogen delivery. Remarkably, replication‐competent VSV_MT_‐S caused no pathogenicity and elicited potent mucosal immunity via intranasal administration, highlighting its potential as a mucosal COVID‐19 vaccine.

## Graphical abstract

The VSV_MT_‐S2P vaccine candidate, featuring a SARS‐CoV‐2 pre‐fusion state spike protein mutant, is safe in healthy and immunocompromised animals. With only single inoculation via various routes of administration, VSV_MT_‐S2P can stimulate effective and comprehensive immune responses in vivo, especially via the intramuscular route. The single‐cycle Vesicular Stomatitis Virus can be a promising vaccine platform for SARS‐CoV‐2 immunogen delivery.

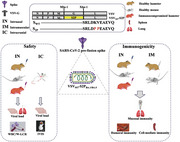

## 1 Introduction

The continuous evolution of SARS‐CoV‐2 has result in the emergence of numerous variants of concern (VOCs), each demonstrating distinct infection patterns, including variations in angiotensin‐converting enzyme 2 (ACE2) receptor usage, transmissibility, and immune evasion capabilities. Among these, the Omicron subvariants BA.5 and XBB.1.5 exhibit significant immune escape potential compared to earlier strains,^[^ ^1^ ^]^ with heightened transmissibility that poses challenges for vaccine development. Several strategies and platforms have been employed in global vaccination campaigns, including protein subunits, inactivated viruses, RNA‐based vaccines, and viral vectors. Given the strong respiratory transmission capacity of the Omicron lineage, the next generation of COVID‐19 vaccines may prioritize fast‐acting mucosal vaccines delivered via a single shot.

Currently, live attenuated viruses or viral vectors are considered the most effective strategies for inducing mucosal immunity against respiratory pathogens. The adenovirus type 5 (Ad5)‐ based intranasal COVID‐19 vaccine has been clinically tested.^[^ ^2^ ^]^ Intranasal administration of the ChAdOx1‐S adenoviral vector vaccine has been shown to elicit superior mucosal immunity compared to intramuscular administration.^[^ ^3^ ^]^ Deng et al. developed an intranasal vaccine candidate using a live attenuated influenza virus (LAIV) encoding the receptor‐binding domain (RBD) of the SARS‐CoV‐2 spike protein.^[^ ^4^ ^]^ Vesicular stomatitis virus (VSV) is another well‐established viral vector extensively studied and utilized in vaccine development. Several VSV‐vectored COVID‐19 vaccine candidates, constructed primarily from replication‐competent wild‐type VSV encoding the spike protein, have been developed.^[^ ^5^ ^]^ To date, at least two VSV‐based vaccine candidates have entered clinical trials: IIBR‐100 (https://clinicaltrials.gov/ct2/show/NCT04990466) and V590, the later produced by Merck. However, the V590 vaccine failed to induce a robust humoral response in Phase I clinical trials, possibly be due to intramuscular administration.^[^ ^6^ ^]^ Our previous research demonstrated that a mutant VSV (VSV_MT_) with triple mutations (S226R, V221F, and ∆M51) in its matrix protein (M) is highly attenuated in animal models.^[^ ^7^ ^]^ When VSV_MT_‐vectored SARS‐CoV‐2 spike proteins from the prototype WA1 and VOCs (Delta and Omicron) were administered intranasally, robust immune responses, particularly mucosal immunity, were observed in hamster and hACE2 transgenic mouse models.^[^ ^8^ ^]^

The immunocompromised population represents a vulnerable demographic in the context of the COVID‐19 pandemic. Their weakened immune systems may lead to reduced vaccine responsiveness, placing them at higher risk for infection and severe disease. Thus, the efficacy and safety of next‐generation vaccines in immunocompromised individuals are of particular concern.

One promising approach for VSV‐vector‐based vaccines is the use of recombinant VSV lacking the G glycoprotein, allowing only a single round of replication.^[^ ^9^ ^]^ This replication‐defective VSV can be engineered to express high levels of foreign antigens.^[^ ^9b^ ^]^ Notably, cellular immune responses to the HIV envelope protein generated by the single‐cycle VSV vector were superior to those induced by the replicative VSV vector.^[^ ^9a^ ^]^ Additionally, the prefusion‐stabilized SARS‐CoV‐2 spike protein (S), modified by two proline substitutions at residues K986 and V987, has proven to be a superior immunogen compared to its wild‐type counterparts.^[^ ^10^ ^]^ In this study, we constructed a single‐cycle VSV with its glycoprotein gene (*G*) replaced by the *S2P* gene, aiming to develop a novel VSV‐vectored SARS‐CoV‐2 mucosal vaccine. We investigated the safety and immunogenicity of a single immunization with VSV_MT_‐S2P via intranasal (IN) or intramuscular (IM) administration, comparing these responses to those generated by the replication‐competent VSV_MT_‐vectored spike protein.

## 2 Results

### 2.1 Construction of a Single‐Cycle VSV_MT_ Vector Encoding the Prefusion State Spike protein of SARS‐CoV‐2 (S2P)

**Figure 1:**
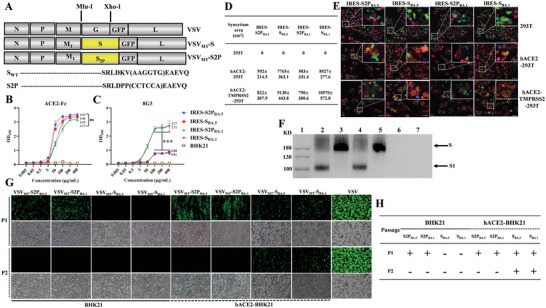
Generation of a non‐propagating VSV_MT_ vector expressing the prefusion stabilized spike protein of SARS‐CoV‐2. The spike protein (S) was engineered with two proline substitutions at residues K986 and V987. Additionally, 21 amino acids were deleted from the C‐terminal region of the spike protein (S_∆21_2P). **A**) Schematic representation of the recombinant VSV constructs expressing the spike (S) protein. The parental VSV_MT_ genome encodes nucleoprotein (N), phosphoprotein (P), glycoprotein (G), matrix protein (M), and RNA polymerase (L). The mutant M protein (MT) carries three specific mutations (M51 deletion, V221F, and S226R). By utilizing *MluI* and *XhoI* restriction enzyme digestion, the *VSV G* protein gene was replaced with either the S_∆21_ protein or the S_∆21_2P mutant from the Omicron subvariants BA.1 or BA.5. The *GFP* reporter gene was inserted between the *G* and *L* genes in the VSV genome. B and C) Intact antigenicity of the S_∆21_2P proteins. S_∆21_2P of both BA.1 and BA.5 subvariants were expressed in BHK21 cells, with the full‐length S proteins from BA.1 and BA.5 serving as controls. The binding activity of human ACE2 to the S_∆21_2P and full‐length S proteins was assessed across a concentration range of 5–200 µg mL^−1^ using a cell‐based ELISA assay B). The antigenicity of S_∆21_2P and full‐length S proteins was further characterized using the BA.1‐specific antibody 8G3 C). D and E) Syncytia formation mediated by the S or S_∆21_2P proteins. 293T cells, transfected to express the GFP reporter along with S_∆21_2P or full‐length S protein from BA.1 or BA.5, were cocultured with cells expressing hACE2 and mCherry, with or without TMPRSS2. Cells were stained with Hoechst (blue), and 24 h later, syncytia (appearing yellow or orange) were visualized using confocal microscopy E); The average syncytium area (µm^2^) was quantified using Image J software D). F) Western blot analysis of rVSV viruses using a spike protein‐specific antibody. VeroE6 cells were infected with VSV_MT_‐S2P or VSV_MT_‐S at an MOI of 1. Cells were harvested and lysed 24 h post‐infection. Lane 1: protein ladder; lane 2–5: lysates from cells infected with VSV_MT_‐S2P_BA.1_, VSV_MT_‐S_BA.1_, VSV_MT_‐S2P_BA.5_, and VSV_MT_‐S_BA.5_ respectively; lane 6: lysates from cells infected with VSV; lane 7: mock control. (G and H) Cellular tropism of VSV_MT_‐S2P and VSV_MT_‐S. hACE2‐BHK21 or BHK21 cells were infected with rVSV_MT_‐S2P, VSV_MT_‐S, or rVSV‐GFP viruses at an MOI of 1. 24 h post‐infection, the supernatants were collected, and the viral progeny were used to infect fresh hACE2‐BHK21 or BHK21 cells. Infected cells were observed under a fluorescent microscope G). H) Summary of viral tropism and replication of rVSVs in cells with or without hACE2 expression. “+”: fluorescence positive; “‐”: fluorescence negative. P1 and P2 represent the first and second rounds of infection, respectively. All the above data are presented as mean ± SD (n = 3). Statistical significance was determined using an unpaired two‐tailed *t*‐test: ns, *p* > 0.05; ***p < 0.001.

The SARS‐CoV‐2 spike protein, with two proline substitutions (S2P) at residues K986 and V987 in the S2 subunit, has been shown to be a superior immunogen.^[^ ^11^ ^]^ In this study, we constructed a replication‐incompetent vector encoding S_∆21_2P, based on an attenuated VSV with triple mutations in the M protein (VSV_MT_‐S2P). In this construct, the VSV glycoprotein gene (*G*) was replaced by the S_∆21_2P gene (**Figure** **1**A). In addition, to facilitate the efficient incorporation of the S2P protein into the VSV envelope, we deleted 21 amino acids from its carboxyl terminal, creating the S_∆21_2P variant.^[^ ^8^, ^12^ ^]^ VSV recombinants lacking the *G* glycoprotein gene can be propagated in cell lines expressing the VSV G protein.

BA.1 was the first Omicron subvariant, while BA.5 is a more recent subvariant known for its greater immune escape potential compared to earlier strains.^[^ ^13^ ^]^ To confirm the intact antigenicity of S_∆21_2P, we transfected the mutant genes of BA.1 or BA.5 into BHK21 cells, using the parental S genes as controls. The expression of the spike proteins on the cell surface was assessed via flow cytometry to ensure consistent protein levels (data not shown). The binding interactions between S_∆21_2P or full‐length S proteins and human ACE2, as well as spike protein‐specific monoclonal antibodies, were measured using cell‐based ELISA assays.

As shown in Figure 1B, within the concentration range of 5–400 µg mL^−1^, the binding activity of both S_∆21_2P and full‐length S proteins with hACE2 increased as the concentration of hACE2‐Fc increased. No significant difference was observed in the binding activity between the S protein and its S_∆21_2P mutant when interacting with hACE2‐Fc (*p* > 0.05) (Table S1, Supporting Information). Thus, the S_∆21_2P mutant retains comparable binding activity with the hACE2 receptor as the full‐length S protein.

The antigenicity of the S2P protein was further evaluated using the 8G3 antibody, which specifically recognizes the S protein of the BA.1 subvariant. As shown in Figure 1C, at concentrations of 100–400 µg mL^−1^, significant differences were detected in the binding activities of 8G3 with the S proteins or the S_∆21_2P mutants of BA.1 and BA.5 (*p* < 0.001). When the 8G3 concentration exceeded 100 µg mL^−1^, its binding activity with S_BA.1_ and S_∆21_2P_BA.1_ reached saturation. At 400 µg mL^−1^, the mean optical density in 450 nm (OD_450_) values for S_BA.1_ and S_∆21_2P_BA.1_ were 2.77 and 2.71, respectively, whereas the mean OD_450_ values for S_BA.5_ and S_∆21_2P _BA.5_ were only 0.88 and 0.81, respectively (Figure 1C, Table S1, Supporting Information). These differences are attributed to amino acid sequence variations between the S_BA.1_ and S_BA.5_ strains. Importantly, the two proline substitutions in the S_BA.1_ protein did not affect its binding activity with the 8G3 antibody. Therefore, the S_∆21_2P mutant retains its original antigenicity and demonstrates potential as an antigen for developing replication‐incompetent VSV‐based vaccines.

SARS‐CoV‐2 can spread to neighboring host cells by forming syncytia, a process triggered by the interaction between viral S proteins and hACE2 receptors on adjacent cells. This phenomenon is a hallmark of severe COVID‐19 cases. The ability of S_∆21_2P and the full‐length S protein to induce syncytia was tested using an in vitro coculture system with dual‐color fluorescent protein labeling.^[^ ^14^ ^]^ In this system, 293T cells were transiently transfected with plasmids encoding either S_∆21_2P or the full‐length *S* gene, along with the *green fluorescent protein* (*GFP*) gene. Another set of 293T cells was transfected with plasmids encoding *hACE2* and *mCherry*, with or without the *transmembrane protease serines* (*TMPRSS2*) gene. Upon cellular fusion, cocultured cells expressing GFP or mCherry produced an orange fluorescence signal, and syncytia formation was quantified after 24 h. In cocultures of 293T cells expressing either S_∆21_2P or the full‐length S protein with 293T cells expressing hACE2, only the aggregation of red donor and green recipient cells was observed, with no yellow or orange‐colored syncytia detected. However, syncytia were observed in hACE2‐expressing 293T cells cocultured with cells expressing the S proteins of BA.1 or BA.5 (Figure 1D,E). These syncytia were significantly larger than those formed in cocultures of hACE2‐293T cells with cells expressing S_∆21_2P of BA.1 or BA.5 (*p* < 0.01). Notably, enhanced syncytia formation was observed when hACE2‐293T cells coexpressed the protease TMPRSS2 with BA.1 and BA.5 S proteins (Figure 1E). These results demonstrate that the SARS‐CoV‐2 spike protein can induce syncytium formation in neighboring hACE2‐expressing cells, with TMPRSS2 further enhancing this process. In contrast, the fusogenicity of S2P among susceptible cells was abrogated due to the prefusion‐stabilized conformation of the S2P protein.

As described in the “Methods” section, replication‐defective VSV was rescued using VSV_MT_. By replacing the *G* gene with either the S_∆21_2P_BA.1_ or S_∆21_2P_BA.5_ gene in the VSV genome, we generated single‐cycle viruses that were propagated in cells expressing the VSV G protein (VSV_MT_‐S2P_BA.1_ or VSV_MT_‐S2P_BA.5_). To confirm the successful rescue of VSV_MT_‐S2P viruses, VeroE6 cells were infected with VSV_MT_‐S2P_BA.1_ or VSV_MT_‐S2P_BA.5_ at a multiplicity of infection (MOI) of 1, with VSV_MT_‐S_BA.1_ and VSV_MT_‐S_BA.5_ used as controls. Western blot analysis with antibodies specific to the SARS‐CoV‐2 S1 domain revealed two bands (180 and 110 kDa) in cells infected with VSV_MT_‐S2P_BA.1_ or VSV_MT_‐S2P_BA.5_. In contrast, only a 180 kDa band was detected in cells infected with VSV_MT_‐S_BA.1_ or VSV_MT_‐S_BA.5_ (Figure 1F). These findings confirm that the S_∆21_2P protein is correctly expressed and processed by furin in cells infected with VSV_MT_‐S2P_BA.1_ or VSV_MT_‐S2P_BA.5_.

To investigate viral tropism and replication characteristics, we tested VSV_MT_‐S2P viruses in BHK21 cells with and without the hACE2 receptor. Replication‐competent VSV_MT_‐S viruses were used as controls. As shown in Figure 1G, VSV_MT_‐S viruses could infect BHK21‐hACE2 cells but not BHK21 cells effectively lacking the hACE2 receptor. In contrast, VSV_MT_‐S2P viruses were able to infect both BHK21 and BHK21‐hACE2 cells due to the G protein in their envelope. However, as shown in Figure 1G, no GFP signal was detected in fresh cells inoculated with progeny VSV_MT_‐S2P viruses, regardless of hACE2 expression. Similar results were observed in Huh7 cells, a human cell line expressing the hACE2 receptor (Figure S1, Supporting Information). These findings indicate that VSV_MT_‐S2P_BA.1_ and VSV_MT_‐S2P_BA.5_ viruses can infect cells with or without the hACE2 receptor, but the viruses are unable to replicate in the infected cells. As described by de Haan et al.,^[^ ^15^ ^]^ the genetic stability of the *S2P* gene in VSV_MT_‐S2P was confirmed by passaging viral stocks and identifying random plaques in BHK‐G cells. All plaques selected from passage 10 of each viral stock retained the K986 and V987 proline substitutions (S2P) (Figure S2, Supporting Information). Additionally, in cells lacking the VSV G protein, VSV_MT_‐S2P viruses collected from plaques could not be serially passaged (data not shown).

Based on these results, we successfully rescued VSVs encoding the S2P proteins of Omicron subvariants BA.1 and BA.5, with 21 amino acids deleted from their carboxyl terminal. These non‐replicating viruses can infect a broad range of cells due to the presence of the G protein in their viral envelope, but they are unable to propagate within the infected cells.

### 2.2 No Significant Toxicity Observed in Healthy Hamsters Administrated Single‐Cycle VSV_MT_‐S2P Viruses

**Figure 2:**
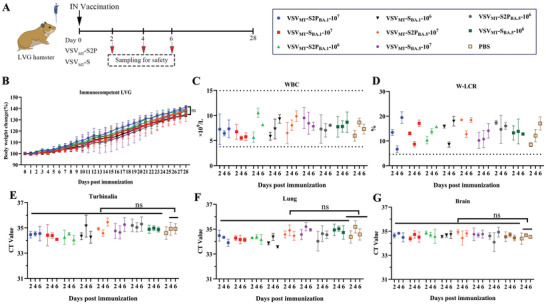
Safety evaluation of VSV_MT_‐S2P and VSV_MT_‐S viruses in hamsters. A) Experimental design for safety evaluation. Female hamsters were divided into groups as outlined in Table 1 (n = 12 each group) and inoculated with either VSV_MT_‐S2P or VSV_MT_‐S at doses of 1 × 10^7^ or 1 × 10^6^ PFU per hamster via intranasal administration. A PBS buffer served as the control. Body weight loss was monitored daily for up to 28 days post‐inoculation (d.p.i.). Sera and organs, including nasal turbinates, lungs, and brains, were collected on 2, 4, and 6 d.p.i. for further analysis (n = 3 each group). B) Body weight changes in inoculated hamsters over the course of the study. C) Total white blood cell (WBC) counts in inoculated hamsters. D) Ratio of large white blood cells (WBC‐large cell ratio, W‐LCR). (E‐G) *VSV N* gene expression was quantified by RT‐qPCR in the nasal turbinates E), lungs F), and brains G) collected on days 2, 4, and 6 d.p.i. All the above data are presented as mean ± SD (n = 3). Statistical significance was assessed using two‐way ANOVA with multiple comparisons: ns, *p* > 0.05.

The study initially assessed the pathogenicity of VSV_MT_‐S2P viruses (VSV_MT_‐S2P_BA.1_ or VSV_MT_‐S2P_BA.5_) in healthy hamsters (**Figure** **2**A). Intranasal administration, commonly used for mucosal vaccine development against SARS‐CoV‐2, is also the most sensitive route for evaluating the pathogenicity of VSV.^[^ ^16^ ^]^ A dose of 1 × 10^7^ plaque‐forming units (PFU) was the maximum concentration achievable for the prepared virus in a 100 µL volume. Accordingly, specific pathogen‐free (SPF) hamsters were divided into groups (**Table** **1**) and inoculated intranasally with either 1 × 10^7^ or 1 × 10^6^ PFU/100 µL of VSV_MT_‐S2P viruses per hamster. Replication‐competent VSV_MT_‐S viruses were also included as controls. To evaluate viral toxicity, several indicators were monitored, including animal body weight, white blood cell (WBC) counts, white blood cell large cell ratio (W‐LCR), and viral loads in various organs, such as the turbinate, lungs and brain.

**Table 1:** Groups of LVG hamster inoculated by VSV_MT_‐S2P or VSV_MT_‐S viruses for evaluations of safety and immunogenicity.

Animal	Inoculum	Routes		Immunization dose	
Safety in LVG hamster^c)^	VSV_MT_‐S2P_BA.5_	IN^a)^		10^6^ PFU	10^7^ PFU
	VSV_MT_‐S2P_BA.1_	IN		10^6^ PFU	10^7^ PFU
	VSV_MT_‐S_BA.5_	IN		10^6^ PFU	10^7^ PFU
	VSV_MT_‐S_BA.1_	IN		10^6^ PFU	10^7^ PFU
	PBS	IN		NA	
Immunogenicity in LVG hamster^d)^	VSV_MT_‐S2P_BA.5_	IN	IM^b)^	10^5^ PFU	10^6^ PFU
	VSV_MT_‐S2P_BA.1_	IN	IM	10^5^ PFU	10^6^ PFU
	VSV_MT_‐S_BA.5_	IN	IM	10^5^ PFU	10^6^ PFU
	VSV_MT_‐S_BA.1_	IN	IM	10^5^ PFU	10^6^ PFU
	PBS	IN	IM	NA	

Female SPF hamsters were inoculated with VSV_MT_‐S2P in 100 µL PBS or mock treated with PBS.

^a)^ IN: intranasal inoculation;

^b)^ IM: intramuscular injection;

^c)^ Female SPF hamsters were inoculated with 10^6^ or 10^7^ PFU VSV_MT_‐S2P in 100 µL PBS via intranasal route. With PBS as the mock control. Each group has 12 hamsters. At 2, 4, and 6 d.p.i., 3 hamsters in each group were euthanized respectively with nasal turbinate, lung, and brain tissues harvested for viral load testing;

^d)^ Female SPF hamsters were inoculated with 10^5^ or 10^6^ PFU VSV_MT_‐S2P in 100 µL PBS via intranasal or intramuscular route for immunogenicity evaluation. Each group has 3 hamsters;

NA. not applicable.

Body weight was monitored daily for 28 days post‐inoculation (d.p.i.). As shown in Figure 2B, in both the 1 × 10^7^ and 1 × 10^6^ PFU group, the body weight of hamsters treated with VSV_MT_‐S2P_BA.1_ or VSV_MT_‐S2P_BA.5_ increased post‐inoculation, with weight gain reaching ≈40% at 28 d.p.i., similar to the PBS control group. No significant differences in body weight gain were observed between the groups (*p* > 0.05). Furthermore, no adverse effects on weight gain were observed in hamsters treated with VSV_MT_‐S viruses, even at the high dose of 1 × 10^7^ PFU. These results are consistent with our previous findings.^[^ ^8^ ^]^

As critical indicators of inflammation and immune response, WBC counts and W‐LCR were assessed in hamsters inoculated with VSV_MT_‐S2P and VSV_MT_‐S viruses. In healthy hamsters, normal WBC counts range between 4 × 10^9^/L^−1^ and 15 × 10^9^/L^−1^.^[^ ^17^ ^]^ In both the 1 × 10^7^ and 1 × 10^6^ PFU VSV_MT_‐S2P groups, WBC counts fluctuated within the normal ranges at 2, 4, and 6 d.p.i., comparable to those in the PBS group. Similarly, no abnormalities in WBC counts were observed in the replication‐competent VSV_MT_‐S virus group (Figure 2C). In healthy LVG hamsters, the normal W‐LCR range is 4% to 50%.^[^ ^17b^ ^]^ In animals inoculated with VSV_MT_‐S2P_BA.1_ or VSV_MT_‐S2P_BA.5_, W‐LCR levels fluctuated between 5% and 20%, again comparable to the PBS group. Similar trends were observed in W‐LCR counts in animals treated with VSV_MT_‐S viruses (Figure 2D). Therefore, neither the replication‐incompetent nor replication‐competent VSV‐based vaccine candidates demonstrated any adverse effects on the inflammation or immune states of healthy hamsters, as indicated by WBC and W‐LCR counts.

To further assess toxicity, animals were euthanized, and tissue samples were collected to measure viral burden in the nasal turbinate, lungs, and brain at 2, 4, and 6 d.p.i. No viral presence was detected in the collected tissues of the rVSV‐treated hamsters using plaque assays (data not shown). Additionally, viral RNA was detected in the tissues using quantitative RT‐PCR with primers targeting a sequence within the VSV nucleocapsid (*N*) gene. The cycle threshold (Ct) values for VSV_MT_‐S2P and VSV_MT_‐S groups showed no significant differences compared to the PBS group (*p* > 0.05) (Figure 2E–G, Table S2, Supporting Information). These results indicate that intranasal administration of both VSV_MT_‐S2P and VSV_MT_‐S viruses does not lead to viral residue in the target organs of hamsters.

In summary, no significant toxicity was observed in healthy hamsters inoculated with VSV_MT_‐S2P or VSV_MT_‐S viruses, even at the high dose of 1 × 10^7^ PFU via the intranasal route.

### 2.3 Immune Response Generation by Single‐Cycle VSV_MT_‐S2P in Hamsters

**Figure 3:**
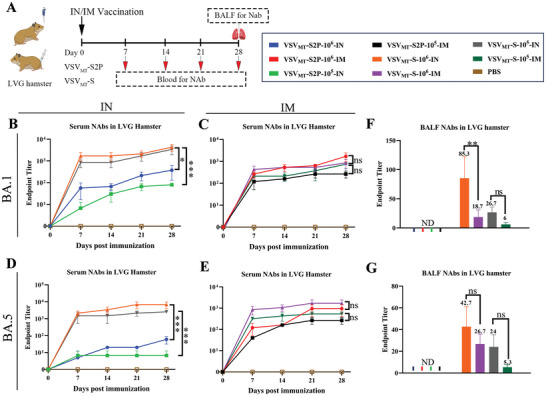
Immunogenicity of VSV_MT_‐S2P and VSV_MT_‐S in healthy hamsters. A) Schematic representation of the vaccination protocol in healthy female hamsters (n = 3 each group). Hamsters were inoculated with VSV_MT_‐S2P or VSV_MT_‐S at doses of 1 × 10^6^ and 1 × 10^5^ PFU per hamster via intranasal or intramuscular administration, with PBS buffer serving as the control. Serum samples were collected every 7 days, and bronchoalveolar lavage fluids (BALF) was collected at 28 days post‐inoculation (d.p.i.). Neutralizing antibody (NAb) titers in the serum and BALF were measured using VSV‐S_BA.1_ and VSV‐S_BA.5_ respectively. The titers were expressed as the reciprocal of the highest antibody dilution that achieved 100% inhibition of the cytopathic effect. B and C) Kinetic curves of NAb titers in the sera of hamsters inoculated with VSV_MT_‐S2P_BA.1_ or VSV_MT_‐S_BA.1_ via intranasal B) or intramuscular routes C). D and E) Kinetic curves of NAb titers in the sera of hamsters inoculated with VSV_MT_‐S2P_BA.5_ or VSV_MT_‐S_BA.5_ via intranasal D) or intramuscular routes E). F) NAb titers in BALF from hamsters immunized with VSV_MT_‐S2P_BA.1_ or VSV_MT_‐S_BA.1_ via intranasal or intramuscular routes. G) NAb titers in BALF from hamsters immunized with VSV_MT_‐S2P_BA.5_ or VSV_MT_‐S_BA.5_ via intranasal or intramuscular routes. All the above data are presented as mean ± SD (n = 3). Statistical significance was determined using two‐way ANOVA with multiple comparisons. ns, *p* > 0.05; *p < 0.05; ***p < 0.001. UD: undetectable.

Based on the previous results, the safety of VSV_MT_‐S2P viruses was confirmed. Subsequently, their immunogenicity was evaluated in healthy hamsters using two doses (1 × 10^5^ and 1 × 10^6^ PFU) administered via intranasal (IN) or intramuscular (IM) routes. The results were compared to those generated by replicative VSV_MT_‐S viruses. Blood samples were collected every 7 days until 28 d.p.i., and bronchoalveolar lavage fluids (BALF) was collected at 28 d.p.i. to assess antibody titers (**Figure** **3**A, Table 1).

As shown in Figure 3B–E and Table S3 (Supporting Information), the humoral neutralizing antibody (NAb) titers induced by the recombinant VSVs were dependent on time, dose, and administration route. In the VSV_MT_‐S2P groups, sera NAbs were detectable as early as 7 d.p.i. via both IN and IM routes, peaking ≈21 d.p.i. Interestingly, VSV_MT_‐S2P viruses elicited stronger humoral NAb responses through the IM route compared to the IN route. Conversely, replicative VSV_MT_‐S viruses induced more robust humoral NAb responses via the IN route than the IM route (Figure 3B–E, Table S3, Supporting Information). Notably, via the IM route, VSV_MT_‐S2P viruses generated similar levels of sera NAbs as the VSV_MT_‐S viruses (Figure 3C,E, Table S3, Supporting Information). However, through the IN route, replication‐competent VSV_MT_‐S viruses produced significantly higher levels of sera NAbs compared to VSV_MT_‐S2P viruses (Figures 3B,D, Table S3, Supporting Information) (*p* < 0.01).

The presence of NAbs in BALF is a critical indicator of mucosal immunity in animals. In this study, BALF was collected by flushing the lungs with 1 mL of PBS. As shown in Figure 3F,G, no NAbs were detected in the BALF of hamsters inoculated with VSV_MT_‐S2P viruses, regardless of the route of administration (IN or IM). In contrast, significant levels of NAbs were detected in the BALF of hamsters inoculated with replicative VSV_MT_‐S viruses, particularly via the IN route (Figure 3F,G). At a dose of 10^6^ PFU of VSV_MT_‐S_BA.1_, the mean NAb titer in BALF reached 85.3 via the IN route, significantly higher than the 18.7 observed via the IM route. Similarly, in BALF samples from hamsters administered 10^6^ PFU of VSV_MT_‐S_BA.5_, the mean titers were 42.7 via the IN route and 26.7 via the IM route. Thus, effective BALF NAbs were detected in hamsters that administered replicative VSV_MT_‐S viruses but not in those given VSV_MT_‐S2P viruses. These results align with previous findings regarding VSV_MT_‐vectored spike proteins from the prototype WA1 and VOCs of Delta and Omicron.^[^ ^8^ ^]^

In conclusion, the single‐cycle VSV_MT_ vector encoding the S2P protein elicited significant NAb responses in serum but not in BALF following a single dose of inoculation. In contrast, the replicative VSV_MT_‐vectored S protein induced strong humoral and mucosal immune responses, particularly through the IN route.

### 2.4 Safety and Immunogenicity of Single‐Cycle VSV_MT_‐S2P Viruses in Mice Following Nasal Administration

As demonstrated in the previous studies, the tropism of VSV_MT_‐S2P is determined by the VSV G proteins, not the SARS‐CoV‐2 spike protein, which represents a key advantage of the VSV_MT_‐S2P viruses. To eliminate potential variations in immune responses across different animal models, the safety and immunogenicity of the VSV_MT_‐S2P viruses were assessed in BALB/c mice.

**Figure 4:**
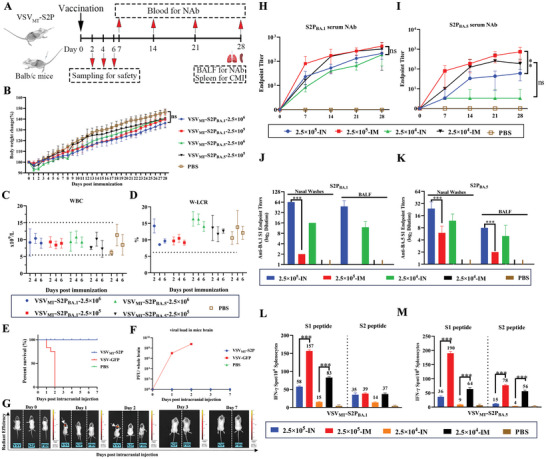
Safety and immunogenicity of VSV_MT_‐S2P in healthy Balb/c mice. A) Experimental design for safety and immunogenicity evaluation in female Balb/c mice. For the safety assessment, adult Balb/c mice were inoculated with VSV_MT_‐S2P at doses of 2.5 × 10^6^ and 2.5 × 10^5^ PFU per mouse via the intranasal route, with PBS as the control (n = 12 each group, 3 female mice in each group at indicated times were euthanized for tissues collecting). Body weight loss was monitored daily for 28 days post‐infection (d.p.i.). Sera were collected at 2, 4, and 6 d.p.i. B) Body weight changes in inoculated mice. C) Total white blood cell (WBC) counts. D) WBC‐large cell ratio (W‐LCR). To assess viral neurovirulence, sixteen‐day‐old suckling mice were intracranially injected with 1500 PFU of VSV_MT_‐S2P_BA.5_ or VSV‐GFP, with PBS as the mock control (n = 15 each group). Mice were monitored daily. E) Survival curve of sucking mice. F) Kinetics of viral replication in the brain. G) IVIS imaging for viral detection. For the immunogenicity assessment, female Balb/c mice were inoculated with VSV_MT_‐S2P at doses of 2.5 × 10^5^ and 2.5 × 10^4^ PFU via intranasal or intramuscular routes, with PBS as the control (n = 3 each group). Sera were collected every 7 days. Bronchoalveolar lavage fluids (BALF), nasal wash, and spleen samples were collected at 28 d.p.i. Neutralizing antibody (NAb) titers were measured using VSV_MT_‐S_BA.1_ and VSV_MT_‐S_BA.5_, expressed as the reciprocal of the highest antibody dilution yielding 100% inhibition of cytopathic effect. (H and I) NAb titers of sera samples from mice inoculated with VSV_MT_‐S2P_BA.1_ H) or VSV_MT_‐S2P_BA.5_ I). J and K) Anti‐spike IgA antibody assay. IgA antibodies in nasal wash or BALF samples from mice vaccinated with VSV_MT_‐S2P_BA.1_ J) or VSV_MT_‐S2P_BA.5_ K) were detected using ELISA. For cellular‐mediated immune responses (CMI), interferon‐gamma (IFN‐γ) levels were measured using an ELISPOT assay as described in the “Methods” section. Splenectomies were performed on mice sacrificed at 28 d.p.i following intranasal or intramuscular vaccination with VSV_MT_‐S2P. CMI responses induced by VSV_MT_‐S2P_BA.1_ L) or VSV_MT_‐S2P_BA.5_ M) were tested using a panel of epitopes from the S1 or S2 regions of the SARS‐CoV‐2 spike protein. Results are presented as mean ELISPOTs per million splenocytes, subtracting any background ELISPOTs from unpulsed mock controls. All the above data are shown as mean ± SD (n = 3). Statistical significance was determined using two‐way ANOVA with multiple comparisons: ns, *p* > 0.05; 0.01 < **p < 0.05; ***p < 0.001. The arrow points to the fluorescent region of the brain. VSV: VSV‐GFP, S2P:VSV_MT_‐S2P_BA.5_.

The toxicity of the VSV_MT_‐S2P viruses was first evaluated by intranasal administration of doses of 2.5 × 10^6^ or 2.5 × 10^5^ PFU per adult mouse, with PBS serving as a control. Mice body weights were monitored daily for 28 d.p.i. (**Figure** **4**A). Additionally, total blood WBC and W‐LCR counts, along with tissue viral loads, were measured. The neurovirulence of VSV_MT_‐S2P viruses was also tested in suckling mice via intracranial injection.^[^ ^18^ ^]^

In the VSV_MT_‐S2P groups administered intranasally, body weights increased from 2 d.p.i., with weight gain reaching ≈38% by 28 d.p.i., similar to the PBS group (*p* > 0.05) (Figure 4B). Total WBC and W‐LCR counts were measured at 2, 4, and 6 d.p.i. WBC counts in healthy mice range from 5.4 to 16 × 10^9^/L.^[^ ^19^ ^]^ In the VSV_MT_‐S2P‐administered mice, WBC counts fluctuated between 6 × 10^9^ and 12 × 10^9^/L at 2, 4, and 6 d.p.i., showing no significant difference from the PBS group (*p* > 0.05) (Figure 4C). The W‐LCR ratio in healthy mice typically fall between 8% and 42.9%.^[^ ^19^ ^]^ The W‐LCR values in the VSV_MT_‐S2P group remained within 8% to 20%, similar to the PBS group (*p* > 0.05) (Figure 4D). These findings indicate that intranasal immunization with replication‐incompetent VSV_MT_‐S2P does not cause abnormal WBC or W‐LCR counts. Viral RNA loads in the nasal turbinate, lungs and brain were also measured using qRT‐PCR, with no significant differences observed between the VSV_MT_‐S2P and PBS groups (Table S4, Supporting Information). This suggests that intranasal administration of VSV_MT_‐S2P does not result in viral persistence in these organs.

Suckling mice (16 days old) were monitored daily using an in vivo imaging system (IVIS) following intracranial injection with VSV_MT_‐S2P_BA.5_, VSV‐GFP, or PBS. All mice injected with VSV‐GFP died at 2 d.p.i., while those injected with VSV_MT_‐S2P _BA.5_ or PBS survived (Figure 4E). IVIS imaging confirmed strong fluorescence signals in the brains of the VSV‐GFP groups at 1 and 2 d.p.i., indicating replication of VSV‐GFP (Figure 4G). In contrast, no fluorescence was detected in the VSV_MT_‐S2P_BA.5_ or PBS groups up to 7 d.p.i. Consistent with this, plaque assay showed no virus detected in the VSV_MT_‐S2P_BA.5_ group, while the viral titers in the VSV‐GFP group reached 9 × 10^6^ PFU/whole brain at 1 d.p.i., and 6 × 10^8^ PFU/whole brain at 2 d.p.i. (Figure 4F). These results, along with those in adult and suckling mice, demonstrate that VSV_MT_‐S2P is safe in healthy animals.

To assess the immunogenicity of VSV_MT_‐S2P in mice, doses of 2.5 × 10^4^ or 2.5 × 10^5^ PFU were administered via IN or IM routes. As shown in Figure 4H,I, NAb titers demonstrated a time‐, dose‐, and the route‐dependent manner, similar to observations in healthy hamsters. Mice produced significant levels of humoral NAbs to both VSV_MT_‐S2P_BA.1_ and VSV_MT_‐S2P_BA.5_, with higher titers observed in the IM group compared to the IN group (Figure 4E,F, and Table S5, Supporting Information). Given the role of IgA antibodies in mucosal immunity, we further evaluated IgA responses in nasal washes and BALF samples using ELISA. As shown in Figure 4J,K, IgA titers induces by VSV_MT_‐S2P were dose‐dependent, with the IN route yielding 4–8 fold higher titers than the IM route in both nasal washes and BALF. However, no NAb was detected in BALFs, regardless of the administration route (data not shown).

Cellular immune responses were assessed using ELISPOT at 28 d.p.i. (Figure 4L,M, Table S6, Supporting Information). Both VSV_MT_‐S2P viruses effectively stimulated cellular immune responses through both IM and IN routes, primarily targeting the S1 peptide pool. T cell responses were dose‐ and route‐dependent, with stronger responses observed in the IM group compared to the IN group.

In summary, VSV_MT_‐S2P demonstrated strong safety profiles in non‐hACE2 transgenic mice following intranasal and intracranial inoculation. The virus induced comprehensive immune responses, including humoral and T cell‐mediated immunity, via both IM and IN administration routes. Notably, a robust mucosal IgA response was detected in nasal wash and BALF samples.

### 2.5 VSV_MT_‐S2P Demonstrated No Apparent Toxicity In Immunocompromised Hamsters, Though Immune Responses were Lower and Delayed

CP has been clinically used to treat cancer and as an immunosuppressive agent for autoimmune and immune‐mediated diseases. It is also commonly used to create immunocompromised animal models for studying viral infections and evaluating vaccines.^[^ ^20^ ^]^ In this study, we used a CP‐induced immunosuppressive hamster model to assess the safety and immune efficacy of VSV_MT_‐S2P and VSV_MT_‐S viruses.

To investigate the effect of CP on VSV infection severity in hamsters, animals were assigned to either a PBS control group or one of three CP regimens: 140 mg kg^−1^, 100 mg kg^−1^, and 70 mg kg^−1^ (Figure S4A, Supporting Information).^[^ ^21^ ^]^ Total WBC counts were measured to confirm immunosuppression. In the PBS group, WBC counts remained within the normal range (4–15 × 10^9^ L^−1^). In CP‐treated animals, WBC counts significantly decreased, confirming immunosuppression, with the trend 140 mg kg^−1^ > 100 mg kg^−1^ > 70 mg kg^−1^ (Figure S4B, Supporting Information). The WBC counts of hamsters treated with 140 mg kg^−1^ CP dropped to 2 × 10^9^ L^−1^ by 2 days post‐treatment and further declined to 0.68 × 10^9^ L^−1^ and 0.34 × 10^9^ L^−1^ by 6 and 12 days, respectively. In contrast, the WBC counts of hamsters treated with 70 mg kg^−1^ CP stabilized ≈3.5 × 10^9^ L^−1^ by day 4 post‐treatment, while those treated with 100 mg kg^−1^ CP maintained ≈1.4 × 10^9^ L^−1^ after day 6. WBC counts between 1.0–2.0 × 10^9^ L^−1^ were classified as moderate leukopenia, and counts < 1.0 × 10^9^ L^−1^ indicated severe leukopenia.^[^ ^22^ ^]^ In hamsters treated with 140 mg kg^−1^ CP, intranasal inoculation with VSV_ΔG_‐S or VSV_MT_‐S resulted in significant weight loss or mortality (Figure S4C, Supporting Information). Based on these findings, we selected 100 mg kg^−1^ CP to establish a moderately immunocompromised model for evaluating the safety and immunogenicity of VSV_MT_‐S2P, VSV_MT_‐S, and VSV_ΔG_‐S viruses. VSV_MT_‐S and VSV_ΔG_‐S possess replication‐competent triple‐site mutated M proteins or wild‐type M proteins, respectively.

**Figure 5:**
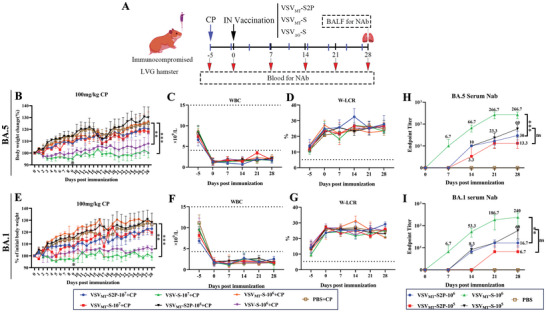
Safety and immunogenicity of VSV_MT_‐S2P compared to VSV_ΔG_‐S in cyclophosphamide‐induced immunocomprised LVG hamsters. A) Experimental design for the safety and immunogenicity evaluations in cyclophosphamide (CP)‐induced immunocompromised female hamsters. Safety assessments were conducted via intranasal administration. Hamsters were inoculated with VSV_MT_‐S2P or VSV_MT_‐S viruses at doses of 1 × 10^7^ and 1 × 10^6^ PFU/hamster, with VSV_ΔG_‐S and PBS serving as controls (n = 3 per group). Body weight loss was monitored daily until 20 days post‐inoculation (d.p.i.). Blood samples were collected on ‐5, 0, 7, 14, 21 and 28 d.p.i. B and E) Body weight changes in immunocompromised hamsters inoculated with rVSVs encoding either the S or S2P proteins of the BA.5 B) or BA.1 E) variants. C and F) White blood cell (WBC) counts in immunocompromised hamsters inoculated with rVSVs encoding either the S or S2P proteins of the BA.5 C) or BA.1 F) variants. D and G) WBC‐large cell ratio (W‐LCR) values in immunocompromised hamsters inoculated with rVSVs encoding either the S or S2P proteins of the BA.5 D) or the BA.1 G) variants. For immunogenicity evaluations, immunocompromised hamsters were inoculated with VSV_MT_‐S2P or VSV_MT_‐S at doses of 1 × 10^6^ and 1×10^5^ PFU/hamster via the intranasal route (n = 3 each group). Serum samples were collected every 7 days, and bronchoalveolar lavage fluids (BALF) was collected at 28 d.p.i. Neutralizing antibody titers in serum and BALF were quantified using VSV_MT_‐S_BA.1_ and VSV_MT_‐S_BA.5_, respectively. Titers are expressed as the reciprocal of the highest antibody dilution yielding 100% inhibition of the cytopathic effect. (H) Neutralizing antibody titers in serum from immunocompromised hamsters vaccinated with VSV_MT_ vectors encoding either the S or S2P proteins of BA.5 via the intranasal route. I) Neutralizing antibody titers in serum from immunocompromised hamsters vaccinated with VSV_MT_ vectors encoding either the S or S2P proteins of BA.1. All the above data are presented as mean ± SD (n = 3). Statistical significance was determined using two‐way ANOVA with multiple comparisons. ns, *p* > 0.05; 0.01 <**p < 0.05; ***p < 0.001.

To assess the safety of various rVSVs in CP‐treated hamsters, two doses (10^7^ and 10^6^ PFU/100 µL) were administered intranasally. To maintain moderate immunosuppression, hamsters received a loading dose of 100 mg kg^−1^ CP followed by maintenance doses of 70 mg k^−1^g at 4‐days intervals (**Figure** **5**A).^[^ ^20a^ ^]^ Animal groups were outlined in Table 3. Body weights were monitored daily until 28 d.p.i., and WBC and W‐LCR counts were measured. Although body weight fluctuated in the VSV_MT_‐S2P group, immunocompromised hamsters still gained weight. By 28 d.p.i., body weight in the PBS group increased by ≈25%, and similar weight gain (≈25%) was observed in high‐dose VSV_MT_‐S2P groups (10^7^ PFU). No significant differences in weight gain were noted between the VSV_MT_‐S2P, VSV_MT_‐S, and PBS groups (*p* > 0.05). In contrast, VSV_ΔG_‐S viruses, which contain wild‐type M proteins, caused significant weight loss in CP‐treated hamsters. For example, hamsters inoculated with VSV_ΔG_‐S_BA.5_ (10^7^ PFU) experienced a 5% weight loss by 9 d.p.i., which was ≈15% lower than the PBS and VSV_MT_‐S2P groups (*p* < 0.05) (Figure 5B). Similarly, no significant weight gain was observed in hamsters inoculated with high‐dose VSV_ΔG_‐S_BA.5_. A similar trend was observed in animals treated with VSV_ΔG_‐S_BA.1_, with a ≈4% weight loss at 9 d.p.i., also significantly lower than the PBS, VSV_MT_‐S2P, and VSV_MT_‐S groups (*p* < 0.05). These results were consistent with the WBC counts, which remained below 2 × 10^9^/L, confirming the immunosuppressed state (Figure 5C,F). Additionally, W‐LCR counts increased following CP treatment (Figure 5D,G). Overall, VSV_ΔG_‐S viruses exhibited toxicity in immunocompromised hamsters, unlike VSV_MT_‐S2P or VSV_MT_‐S.

Next, we assessed the immune responses stimulated by VSV_MT_‐S2P and VSV_MT_‐S viruses in immunocompromised hamsters via intranasal inoculation at doses of 10^6^ and 10^5^ PFU. The immune responses were both time‐ and dose‐dependent. No NAbs were detected in sera from VSV_MT_‐S2P‐inoculated animals at 7 d.p.i., but titers slowly increased by 28 d.p.i. The mean NAb titers induced by VSV_MT_‐S2P_BA.1_ and VSV_MT_‐S2P_BA.5_ were 17 and 30, respectively. In contrast, higher levels of humoral NAb were detected in VSV_MT_‐S group (Figure 5H,I, Table S7, Supporting Information). No NAbs were detected in BALF samples from either VSV_MT_‐S2P‐ or VSV_MT_‐S‐inoculated immunocompromised animals (data not shown).

In summary, in a cyclophosphamide‐induced immunocompromised hamster model with moderate immunosuppression (100 mg kg^−1^ CP), VSV_MT_‐S2P was safe when administered via the sensitive intranasal route. However, the replication‐defective VSV_MT_‐S2P viruses induced relatively low levels of humoral NAbs, compared to those in healthy animals. Notably, VSV_MT_‐S demonstrated no toxicity in immunocompromised hamsters and stimulated effective immune responses with a single intranasal shot, producing sera NAb titers tenfold higher than those induced by VSV_MT_‐S2P viruses.

## 3 Discussion

Although the acute phase of SARS‐CoV‐2 pandemic has subsided, the virus continues to circulate. Multiple vaccines have been developed, playing a critical role in controlling the spread of COVID‐19. However, the rapid transmission of the Omicron lineage and its subvariants through upper respiratory tract has underscored the need for novel vaccines. Wellford et al. highlighted that while systemic antibodies can protect respiratory tissues, they may not be effective in olfactory tissues due to the blood‐brain barrier in the olfactory epithelium.^[^ ^23^ ^]^ This finding suggests that fast‐acting mucosal vaccines, administered in a single dose, should be the focus for the next generation of COVID‐19 vaccines.

Currently, attenuated live viruses and viral vectors remain the most effective strategies for inducing mucosal immunity against respiratory pathogens.^[^ ^24^ ^]^ Amy et al. reported that mucosal administration of VSV_ΔG_‐SARS‐CoV‐2 elicited a stronger immune response than intramuscular injection in hamsters.^[^ ^25^ ^]^ Our previous studies also demonstrated that VSV_MT_ encoding the SARS‐CoV‐2 spike protein could induce comprehensive and potent immune responses, particularly through the intranasal route.^[^ ^8^ ^]^ However, considering that viral vectors limited to a single cycle of replication are subject to less stringent regulatory standards, we developed a replication‐incompetent VSV_MT_ vector as a vaccine platform for SARS‐CoV‐2. In this study, we replaced the *G* protein gene in the VSV_MT_ genome with the *S2P* gene from the Omicron BA.1 or BA.5 strain. To control for variability duo to different animal models and inoculation routes, we tested the vaccine candidates in both hamsters and mice, administered via either intranasal or intramuscular routes.^[^ ^7^, ^8^ ^]^ The vaccine candidates have several notable features. First, the replication‐incompetent VSV_MT_ provides the advantage of being a safer vector while still initiating robust humoral, cellular, and mucosal immunity. This is particularly important for vulnerable populations, such as immunocompromised individuals. Second, because the virion is packaged with VSV G, its tropism is expanded due to the widespread distribution of the VSV G receptor, the low‐density lipoprotein receptor (LDLR).^[^ ^26^ ^]^ This could enhance antigen presentation through multiple inoculation routes, such as intramuscular administration, where skeletal muscle cells express minimal ACE2. In our in vitro studies, with the VSV G protein replaced by the stabilized S2P protein, the VSV_MT_‐S2P viruses were able to infect cells regardless of hACE2 receptor expression. However, since these viruses lack the *G* gene in their genome, progeny viruses were unable to infect other cells.

When administered intranasally, wild‐type VSV can cause neurotoxicity, including hind limb paralysis in healthy animals.^[^ ^16b^ ^]^ In a previous study, wild‐type VSV‐vectored spike protein from the Delta strain (VSV_∆G_‐S Delta) caused pathogenic effects in healthy hamsters via intranasal administration.^[^ ^8^ ^]^ To address safety concerns, this study focused on the intranasal route to evaluate its safety as a potential mucosal vaccine. The VSV_MT_ encoding the *S2P* gene from either the BA.1 or BA.5 strain did not result in significant adverse toxicity in healthy hamsters or mice, even when administered at an extremely high dose of 10^7^ PFU. Moreover, intracranial injections of VSV_MT_‐S2P did not cause neurovirulence, such as viral replication in the brain or mortality in naive suckling mice. Importantly, in immunocompromised animals treated with CP, no toxicity, body weight loss, or detectable viral loads in target organs were observed in animals inoculated with VSV_MT_‐S2P. Similarly, highly attenuated replication‐competent VSV_MT_‐S viruses did not cause toxicity in healthy or immunocompromised hamsters. In contrast, replication‐competent VSVs with the wild‐type M protein (VSV_ΔG_‐S) induced toxicity, including body weight loss, in CP‐treated immunocompromised hamsters. Based on these findings, both the replication‐incompetent VSV_MT_‐based vector and the replication‐competent VSV_MT_ vector show promise as safe and effective platform for developing COVID‐19 vaccine candidates.

Previous studies have demonstrated that the route of administration plays a crucial role in triggering both local and systemic immune responses when using the VSV vector.^[^ ^27^ ^]^ Notably, the VSV‐vectored SARS‐CoV‐2 vaccine candidate, V590, failed to elicit an effective humoral response during Phase I clinical trials, which may be partly attributed to its intramuscular (IM) administration. One possible explanation is the minimal expression of ACE2 receptors on the surface of skeletal muscle cells, potentially reducing the efficiency of VSV‐SARS‐CoV‐2 spike vaccines like V590 when administered intramuscularly. Interestingly, in this study, robust immune responses induced by VSV_MT_‐S2P were observed via both IM and IN administration in healthy hamsters and mice. As a negative‐strand RNA virus, VSV completes a single round of replication in host cells in less than 8 h.^[^ ^28^ ^]^ This rapid replication makes VSV‐based vaccine vectors particularly effective, as demonstrated in their use against Ebola virus (EBOV) in humans, which is essential for controlling fast‐spreading epidemics such as COVID‐19. Importantly, both the VSV_MT_‐S2P and VSV_MT_‐S viruses generated humoral responses in healthy hamsters and mice as early as 7 days post‐inoculation. However, notable differences were observed in the magnitude of the immune responses triggered by VSV_MT_‐S2P and VSV_MT_‐S, including the following: 1. Route‐Dependent Humoral Response: VSV_MT_‐S2P elicited a higher level of NAbs via the IM route compared to the IN route. Conversely, VSV_MT_‐S stimulated more potent humoral NAbs responses via the IN route than the IM route. 2. Comparable NAb Responses via IM Route: When administered intramuscularly, VSV_MT_‐S2P induced NAb levels comparable to those of VSV_MT_‐S in healthy animals, with titers reaching as high as ≈10^3^. However, when administered intranasally, VSV_MT_‐S2P induced a ≈10‐fold lower NAbs titer in sera compared to VSV_MT_‐S. 3. BALF NAb Levels: BALF NAbs are a key indicator of mucosal immunity. In this study, no significant NAb levels were detected in the BALF of hamsters administered VSV_MT_‐S2P at the tested doses. In contrast, considerable levels of BALF NAbs were detected in animals treated with VSV_MT_‐S via the IN route. 4. Anti‐Spike IgA in Nasal Washes: In nasal washes of mice, anti‐spike IgA, reflecting upper respiratory immunity, was stimulated by VSV_MT_‐S2P in a dose‐dependent manner. Titers via the IN route were 4‐ to 8‐fold higher than those via the IM route. 5. T‐Cell Responses: T‐cell responses are critical for preventing the progression of COVID‐19. Using Balb/C mice (which lack hACE2 receptor), the single‐cycle VSV_MT_‐S2P vector generated a more potent cellular immune response via the IM route compared to the IN route. This result is consistent with previous findings for single‐cycle VSV vectors encoding the HIV envelope protein.^[^ ^29^ ^]^ These results highlight the significant differences in immune response patterns between the two viral vectors, likely due to differences in viral tropism and replication characteristics.

Immunocompromised individuals represent a heterogeneous group with varying degree of immune suppression, immune reconstitution, and viral control, placing them at high risk for COVID‐19. CP, an alkylating agent, is widely used in clinical treatments for cancer and autoimmune diseases.^[^ ^30^ ^]^ However, CP administration can disrupt the Th1/Th2 balance and lead to a significant reduction in the absolute counts of T cells and B cells.^[^ ^31^ ^]^ It is also commonly used to establish immunocompromised animal models.^[^ ^21^, ^32^ ^]^ Leukopenia (white blood cell count below 4 × 10^9^ L^−1^) is often regarded as a direct indicator of successful model establishment. Minjeong Nam et al. classified leukopenia into three categories: mild (2.0–4.0 × 10^9^ L^−1^), moderate (1.0–2.0 × 10^9^ L^−1^), and severe (<1.0×10^9^ L^−1^).^[^ ^22^ ^]^ In our study, we used a dose of 100 mg kg^−1^ CP, which resulted in total white blood cell counts decreasing to 1.0–2.0 × 10^9^ L^−1^, indicating moderate leukopenia. In immunosuppressed animals, both the NAb titers and the time to peak immune response induced by VSV_MT_‐S2P were reduced compared to healthy animals. During the first two weeks post‐vaccination, almost no or very low titer of serum NAbs (<1:10) were detected in immunocompromised hamsters. By 21–28 days post‐ infection (dpi), NAb titers in CP‐treated animals reached ≈60, which was 10‐fold lower than those observed in non‐CP‐treated animals. Additionally, although peak NAb titers in CP‐treated hamsters reached levels above 1:200 after VSV_MT_‐S vaccination, thses peaks occurred at ≈21 d.p.i., compared to 7 d.p.i. in healthy animals. Thus, immunosuppressive treatment with CP had a relatively greater impact on the efficacy of the replication‐incompetent VSV‐vectored vaccine candidate compared to the replication‐competent version.

For vaccine development based on viral vectors, there is usually a balance between vector virus attenuation and immunogenicity. VSV‐S2P is very safe and can activate a comprehensive host immune response effectively. Our studies, using both healthy and immunocompromised animal models, also revealed certain limitations of VSV_MT_‐S2P as a vaccine candidate. When administered intranasally, VSV_MT_‐S2P elicited relatively weak immune responses compared to VSV_MT_‐S, particularly in BALF. However, based on previous results with the ChAd‐SARS‐CoV‐2‐S vaccine in mice, which employed a dosage of 10^10^ viral particles,^[^ ^33^ ^]^ it is suggested that a higher dose of VSV_MT_‐S2P may be required to induce stronger BALF NAbs responses in future studies. VSV_MT_‐S2P was found to be safe when administered intranasally at doses as high as 10^7^ PFU. Furthermore, as suggested by Beitari et al.,^[^ ^34^ ^]^ a two‐dose intranasal administration strategy could be an effective way to enhance mucosal immunity with VSV_MT_‐S2P.

Notably, studies conducted in CP‐induced immunocompromised animals provided valuable insights into the VSV_MT_‐S vaccine candidate. Comprehensive evaluations in both healthy and immunocompromised animal models demonstrated that the replication‐competent VSV_MT_‐S vaccine caused no pathogenesis, while effectively stimulating mucosal immunity when administered intranasally. This makes it a promising candidate for a mucosal COVID‐19 vaccine.

## 4 Conclusion

The balance between safety and strong immunogenicity has always been a concern for developing vaccines. The VSV_MT_‐S2P viruses are very safe in health as well as immunosuppressive animals. With only single inoculation via various routes of intranasal and intramuscular administration in healthy animals, VSV_MT_‐S2P viruses can stimulate effective and comprehensive immune responses in vivo, especially via intramuscular route. In addition, it should be pointed out that via intranasal route, VSV_MT_‐S2P induce relatively weak immune responses, especially in BALF. Thus, considering the safeness of VSV_MT_‐S2P, a higher dose of the vaccine candidate might be used in the following study intranasally. Furthermore, a 2‐dose intranasal administration pattern may also be the strategy to improve mucosal immunity by VSV_MT_‐S2P.

## 5 Experimental Section

### Cell Lines and Viruses

Baby hamster kidney cells (BHK‐21) (CCL‐10, ATCC), VeroE6 cells (CRL‐1586, ATCC), 293T cells (ATCC), and Huh‐7 cells were cultured in DMEM medium supplemented with 10% fetal bovine serum (Gibco, USA). All cells were maintained in a humidified atmosphere containing 5% CO_2_ at 37 °C. VSV‐GFP was stored in the laboratory.

### Plasmid Construction

The human codon‐optimized sequences of the SARS‐CoV‐2 spike (S) protein genes from the Omicron subvariants BA.1 and BA.5 (GenBank: OX008556.1; OP984772.1) were cloned into the eukaryotic expression plasmid *p*IRES. The prefusion SARS‐CoV‐2 spike antigen containing two proline substitutions at residues K986 and V987 (S2P) was generated using overlapping PCR based on these constructs and subsequently cloned into *p*IRES. Additionally, the serine protease and hACE2 genes were cloned into *p*IRES using the One Step Seamless Cloning Kit (Yeasen, China).

### Syncytia Formation Assay

Syncytia formation induces by S_∆21_2P (S2P) and full‐length S proteins was assessed using an in vitro coculture system with dual‐color fluorescent protein labeling.^[^ ^14^ ^]^ Briefly, 293T cells were seeded in 12‐well plates and transfected 24 h later with either the S2P or S gene‐expressing plasmids (0.4 µg each), along with GFP plasmids. Concurrently, other 293T cells were transfected with plasmids expressing ACE2 and mCherry, with or without the *TMPRSS2* gene. After 16 h, the transfected cells were cocultured at a 1:1 ratio. Following cell fusion, GFP and mCherry co‐expression produced an orange fluorescence signal. After 24 h, the area of syncytia was quantified using Image J software.

### Recovery of Recombinant VSV_MT_‐S2P

The SARS‐CoV‐2 spike genes from BA.1 and BA.5 strains, along with their S2P mutants, were cloned into the *p*VSV_MT_‐GFP plasmid. To facilitate the efficient incorporation of S2P into the VSV envelope, 21 amino acids were deleted from the carboxyl terminal. As shown in Figure 1A, the *G* gene in the VSV Indiana strain genome was excised using *XhoI* and *MIuI* restriction enzymes and replaced with the *S* or *S2P* genes from BA.1 or BA.5, creating the constructs *p*VSV_MT_‐S2P_BA.1_, *p*VSV_MT_‐S2P_BA.5,_ *p*VSV_MT_‐S_BA.1_, and *p*VSV_MT_‐S_BA.5_. The *GFP* gene was inserted at the *Xho*I and *Nhe*I sites between the VSV *G* and *L* genes of the viral genome.

The recovery of viruses from these plasmids was performed as previously described.^[^ ^8^ ^]^ In brief, BHK‐21 cells were infected for 1 h with recombinant vaccinia virus (vTF7‐3), which expresses T7 RNA polymerase. The infected cells were then co‐transfected with pVSV_MT_‐S2P or pVSV_MT_‐S plasmids, along with the helper plasmids *p*BS‐N, P, L, and G. After 48 h of transfection, the virus‐containing supernatants were filtered through a 0.2 µm filter to remove residual vaccinia virus and passaged onto BHK‐21 cells transfected with pCAGGS‐G (BHK‐G), a plasmid expressing the VSV G protein. After 48 h, the supernatant was collected upon the appearance of cytopathic effects in the cells. The VSV_MT_‐S2P viruses were plaque‐purified and amplified in BHK‐G cells, ensuring that the virions expressed the G protein on their surface, enabling infection. Replication‐defective VSV_MT_‐S2P was titrated on BHK‐G cells. The VSV_MT_‐S viruses were plaque‐purified, amplified, and titrated in VeroE6 cells.

### Genetic Stability of the *S2P* Gene in VSV_MT_‐S2P

To assess the genetic stability of VSV_MT_‐S2P, we conducted a series of analyses including viral stock passaging, plaques purification, gene amplification, and sequencing, as previously described.^[^ ^15^ ^]^ In brief, the Master Seed Virus (MSV) of VSV_MT_‐S2P was serially passaged up to 10 times in BHK21 cells expressing the VSV G protein (BHK‐G). The virus from the 5th and 10th passages was plaque‐purified, and at least 10 viral plaques were randomly selected from each viral stock. Viral RNA was extracted, and the *S2P* gene was amplified through reverse transcription, followed by sequencing. The PCR primers used were: forward, 5′‐GCTTTATCGAGGACCTGCTG‐3′; and reverse, 5′‐GTCACGTGCAGAAACACCAC‐3′. Additionally, the VSV_MT_‐S2P viruses collected from the plaques were serially passaged in BHK21 cells lacking VSV G protein expression. Cells were monitored using fluorescent microscope to observe viral replication and expression.

### Western Blot

VSV_MT_‐S2P and VSV_MT_‐S viruses were identified using Western blotting with an antibody recognizing the SARS‐CoV‐2 spike protein. Briefly, VeroE6 cells were infected with VSV_MT_‐S2P or VSV_MT_‐S viruses at a MOI of 1 for 1 h. After the inoculum was removed, the cells were washed, and fresh growth medium was added to allow the infection to continue for 24 h. The cells were then scraped and lysed in 5 × SDS‐PAGE loading buffer containing 1% PMSF. The lysates were centrifuged at 15, 700×g for 15 min at 4 °C. Proteins were separated on an SDS‐PAGE gel at 110 V for 1 h and transferred to a PVDF membrane. The membrane was incubated with a rabbit anti‐spike antibody (SinoBiological, China) at 4 °C for 12 h. After five washes, the membrane wad incubated with the HRP‐conjugated goat anti‐rabbit IgG antibody at room temperature for 1 h. Following another five washes with PBST, the bands were visualized using ECL regent.

### Neutralization Assay

The neutralization assay was performed following a previously described protocol.^[^ ^35^ ^]^ Briefly, sera from experimental animals were heat‐inactivated at 56 °C for 30 min before testing. The sera were then serially diluted two‐fold, starting at 1:10, in DMEM. BALF samples were similarly diluted two‐fold, starting at 1:2. To determine neutralizing antibody titers, 50 µL of the diluted sera or BALF was mixed with 50 µL of DMEM containing 100 PFU of VSV_ΔG_‐S_Δ21_‐GFP, which encodes the S_Δ21_ spike protein from BA.1 or BA.5. The mixtures were incubated at 37 °C for 1 h before being added to VeroE6 cells cultured in 96‐well plate. After 1 h, the cells were rinsed three times with DPBS and incubated in DMEM containing 2% FBS. At 24 hours post‐infection (h.p.i.), neutralization titers were determined by observing the cytopathic effect under a fluorescence microscope. The titers were recorded as the reciprocal of the highest antibody dilution that achieved 100% inhibition of the cytopathic effect. Neutralization curves were generated using four‐parameter nonlinear regression in GraphPad Prism 8.

### Quantitative Reverse Transcriptase PCR (qRT‐PCR)

All tissues were weighed and homogenized with Zirconia bead in 1 mL PBS. Total RNA was extracted from clarified tissue homogenates using TRIzol Regent (Invitrogen, USA). Briefly, 750 µL of Trizol reagent was mixed with 250 µL of homogenized tissue supernatant. After incubating for 5 min, 200 µL of chloroform was added, and the mixture was vortexed for 15 s. The samples were centrifuged for 15 min at 11 000 ×g, and 300 µL of the upper aqueous phase was collected and mixed with 300 µL of isopropanol. The mixture was stored at −20 °C for 2 h. Following centrifugation for 20 min at 11 000 ×g, the RNA pellete was washed with 75% ethanol. After a final centrifugation, the RNA was dissolved in 20 µL DEPC‐treated water (Sangon, China). The concentration of RNA was detected with NanoDrop 2000 (Thermofisher, USA) and viral cDNA was synthesized using the First Strand RevertAid cDNA Synthesis Kit (Thermo Fisher, USA). qRT‐PCR was performed using the Prmix Pro Taq HS Qpcr SYBR Green II Kit (Acccurate Biology, China). PCR amplification was performed with a thermal cycler (Analytik Jena, Jena, Germany) with initial enzyme activation at 95 °C for 30 s and then 40 cycles of 95 °C for 30 s and 60 °C for 30 s. The primers used to detect *VSV‐N* gene were as follows: forward, 5′‐TGATCGACTTTGGATTGTCTTCTAA‐3′, reverse, 5′‐TCTGGTGGATCTGAGCAGAAGAG‐3′. The threshold value was calculated automatically (it is actually 10 times the standard deviation of the 3–15 cycles of the fluorescence signal). Viral RNA was expressed as *VSV N* gene CT value per milligram.

### Cell‐Based ELISA

Detection of affnity activity of S2P with ACE2‐Fc or 8G3 antibody was performed by a cell‐based ELISA.^[^ ^36^ ^]^ Briefly, BHK21 cells were transfected with 8 µg of the *p*IRES‐S2P_BA.1_, *p*IRES‐S2P_BA.5,_ *p*IRES‐S_BA.1_ or *p*IRES‐S_BA.5_ using Lipofectamine 2000 (Thermofisher, USA). 5 h post‐transfection, the cells were trypsinized, washed with PBS, and then seeded into a 96‐well plate at a density of 1 × 10^5^ cells well^−1^ in 100 uL of DMEM containing 10% FBS. The cells were incubated for 24 h at 37 °C. The wells were washed twice with PBS, and 125 µL of 10% buffered formalin was added to each well for 10 min at room temperature. After this, the wells were washed three times with distilled water (diH_2_O) and dried. Next, 250 µL of 2% BSA (prepared in PBS) was added to each well and incubated for 1 h at 37 °C. Following three washes with diH_2_O, 50 µL well^−1^ of ACE2‐Fc protein or 8G3 antibody at concentration of 0, 0.005, 0.05, 0.5, 5, 50, 100, 200, or 400 µg mL^−1^ (diluted in 1% BSA) were added to the wells and incubated for 2 h at 37 °C. The wells were then washed five times with diH_2_O, and 50 µL well^−1^ of anti‐human IgG‐HRP (diluted 1:5000 in 1% BSA, from Abmart, china) were added and incubated for 1 h at 37 °C. After five washes with diH_2_O, the plate was rinsed with carbonate buffer. Then, 50 µL well^−1^ of TMB substrate solution (Solabio, China) was added and incubated for 20 min at room temperature. The reaction was stopped by adding 25 uL well^−1^ of 4.5N sulfuric acid, and the OD_450_ value was measured using a Microplate Reader AMR‐100 (Allsheng, Hangzhou, china). Affinity curves were generated using four‐parameter non‐linear regression in GraphPad Prism 8.

### Anti‐Spike IgA ELISA

A 96‐well plate was precoated with 2.5 µg mL^−1^ recombinant S1 antigen from either the BA.1 or BA.5 strain (SinoBiological, China) in carbonate buffer solution (0.05 M Na_2_CO_3_, 0.05 M NaHCO_3_) and incubated overnight at 4 °C. The plates were then blocked with 2% BSA in PBS containing 0.25% Tween 20 (PBST) and incubated for 1 h at room temperature. Following this, the plates were washed three times with PBST. BALF or nasal samples were serially diluted two‐fold and added to the plates, which were then incubated for 1 h at 37 °C. After three more washes with PBST, HRP‐conjugated goat anti‐mouse IgA antibody (1:10 000, Abcam, UK) was added to each well and incubated for 1 h at room temperature. The plates were washed three more times, and 100 µL of TMB substrate solution was added to each well, followed by a 30‐min incubation in the dark at room temperature. The reaction was stopped by adding 100 µL of phosphoric acid, and the plates were read at 450 nm using the Microplate Reader AMR‐100 (Allsheng, Hangzhou, China). ELISA titers were calculated using the positive‐negative (P/N) ratio, where the endpoint was defined as the highest dilution with a P/N ratio of ≥2.1.^[^ ^37^ ^]^

### IFN‑γ ELISPOT

Splenectomies were performed on mice administered VSV_MT_‐S2P viruses, which were sacrificed at 28 d.p.i. After anesthesia, the spleen were aseptically removed, washed with sterile PBS, and cut into small pieces using sterile scissors. The tissue was then homogenized, and the splenocytes were transferred to 50 mL conical tubes and centrifuged at 300×g for 5 min. The cell pellets were resuspended in PBS, and the cell suspension was filtered through a sterile 40 µm cell strainer. Murine lymphocytes were isolated according to the manufacturer's protocol (Solarbio, China). The isolated cells were resuspended in 1 mL RPMI‐1640 medium containing 10% FBS and counted for the ELISPOT assay, which was performed according to the manufacturer's protocol (Mabtech, Sweden). Briefly, cells harvested from each mouse were seeded in triplicate at a density of 3 × 10^5^ cells per well into 96‐well plates precoated with an IFN‐γ monoclonal antibody. The cells were stimulated with a 2 µg mL^−1^ peptide pool derived from the SARS‐CoV‐2 spike protein (PP003, SinoBiological, China) at 37 °C in a CO_2_ incubator. Additionally, cells from each mouse group were plated as mock controls (non‐pulsed). After 20 h of incubation, the cell medium was removed, and the plates were extensively washed with PBS containing 0.5% FCS. A biotin‐labeled anti‐IFN‐γ monoclonal antibody (0.5 µg mL^−1^) was then added to each well. The plates were incubated for 90 min at room temperature, followed by additional washes with PBS‐containing 0.5% FCS. Next, 100 µL of streptavidin‐alkaline phosphatase (0.2 µg mL^−1^) was added to each well, and the plates were incubated for 1 h at room temperature before being washed again. A ready‐to‐use substrate solution (BCIP/NBT‐plus) was added at 100 µL well^−1^ to develop the spots. After spot development, the plates were thoroughly rinsed with tap water, dried and the spots were quantified. Any background ELISPOTS observed in the mock wells were subtracted from the experimental groups. The results are expressed as the mean number of ELISPOTs per million cells±standard deviation for each group.

### Animal Experiments

All animal studies were conducted in accordance with protocols approved by the Shanghai Veterinary Research Institute (SV‐20231201‐02) and adhered to ethical guidelines set by Shanghai JiaoTong University. Specific‐pathogen‐free (SPF) female Syrian hamsters and Balb/c mice were obtained from Charles Rivers Laboratories. Virus inoculations were performed under anesthesia induced and maintained with ketamine hydrochloride and xylazine, with all efforts were made to minimize animal suffering.

In healthy female hamsters, the toxicity of VSV_MT_‐S2P or VSV_MT_‐S was evaluated at doses of 10^7^ PFU or 10^6^ PFU in 100 µL of PBS administered via the intranasal route. PBS were used as a mock control. Groups of hamsters (≈g) are detailed in Table 1 (n = 12 each group). To assess viral toxicity, the body weights of hamsters were monitored daily for 28 d.p.i. Blood samples were collected at 2, 4, and 6 d.p.i. for hematology analysis, using WBC count and W‐LCR as indicators. Additionally, 3 hamsters in each group were euthanized at 2, 4, and 6 d.p.i. respectively and animal tissues including nasal turbinate, lung, and brain were harvested for viral load testing. For immunogenicity tests, female hamsters were grouped (n = 3 each group) and inoculated with 10^6^ PFU or 10^5^ PFU of VSV_MT_‐S2P or VSV_MT_‐S in 100 µL of PBS, administered either intranasally (IN) or intramuscularly (IM) (Table 1). Blood samples from immunized hamsters were collected at 7, 14, 21, and 28 d.p.i. for NAbs tests. Additionally, BALF was harvested at 28 d.p.i, after euthanizing the animals for further NAb testing.^[^ ^38^ ^]^

In adult female Balb/c mice, viral toxicity was evaluated with doses of 2.5 × 10^6^ PFU or 2.5 × 10^5^ PFU of VSV_MT_‐S2P in 50 µL of PBS administered intranasally. Groups of mice are outlined in **Table** **2** (n = 12 each group). Body weights were monitored daily for 28 d.p.i. Blood samples were collected at 2, 4, and 6 d.p.i. for hematology analysis, with WBC count and W‐LCR as indicators. In addition, 3 mice in each group were euthanized at 2, 4 or 6 d.p.i respectively and tissues including nasal turbinate, lung and brain were harvested for viral load testing. For immunogenicity testing, female Balb/c mice (n = 3 each group), were inoculated with VSV_MT_‐S2P via the IN or IM routes at doses of 2.5 × 10^5^ PFU or 2.5 × 10^4^ PFU in 50 µL of PBS (Table 2). Blood samples were collected at 7, 14, 21, and 28 d.p.i. for NAb tests. At 28 d.p.i., mice were euthanized, and lungs were flushed with 1 mL of PBS to collect BALF. Nasal washes were obtained by injecting 200 µL of PBS through a catheter into the trachea and collecting the flow‐through from the nares.^[^ ^38^ ^]^

**Table 2:** Groups of Balc/C mice inoculated by VSV_MT_‐S2P or VSV_MT_‐S viruses for evaluations of safety and immunogenicity.

Animal	Inoculum	Routes		Immunization dose
Safety evaluation^c)^	VSV_MT_‐S2P_BA.5_	IN^a)^		2.5 ×1 0^5^ PFU
	VSV_MT_‐S2P_BA.5_	IN		2.5 × 10^6^ PFU
	VSV_MT_‐S2P_BA.1_	IN		2.5 × 10^5^ PFU
	VSV_MT_‐S2P_BA.1_	IN		2.5 × 10^6^ PFU
	PBS	IN		NA
Immunogenicity evaluation^d)^	VSV_MT_‐S2P_BA.5_	IN	IM^b)^	2.5 × 10^4^ PFU
	VSV_MT_‐S2P_BA.5_	IN	IM	2.5 × 10^5^ PFU
	VSV_MT_‐S2P_BA.1_	IN	IM	2.5 × 10^4^ PFU
	VSV_MT_‐S2P_BA.1_	IN	IM	2.5 × 10^5^ PFU
	PBS	IN	IM	NA

Female SPF Balb/c mice were inoculated with VSV_MT_‐S2P at dose of 2.5 × 10^4^, 2.5×10^5^ or 2.5×10^6^ PFU in 50 µL PBS or mock treated with PBS.

^a)^ IN: intranasal inoculation

^b)^ IM: intramuscular injection

^c)^ Female SPF Balb/c mice were inoculated with VSV_MT_‐S2P at dose of 2.5 × 10^5^ or 2.5 × 10^6^ PFU in 50 µL PBS via intranasal route. With PBS as the mock control. Each group has 12 mice. At 2, 4, and 6 d.p.i., 3 mice in each group were euthanized respectively with nasal turbinate, lung, and brain tissues harvested for viral load testing.

^d)^ Female SPF Balb/c mice were inoculated with VSV_MT_‐S2P at dose of 2.5 × 10^4^ or 2.5×10^5^ PFU in 50 µL PBS via intranasal or intramuscular route for immunogenicity evaluation. Each group has 3 mice.

NA. not applicable.

To assess the neurovirulence of rVSVs, 16‐day‐old female mice (n = 15 each group) were injected intracerebrally with VSV_MT_‐S2P_BA.5_, VSV‐GFP, or PBS using a 1 µL Hamilton syringe. Injection were made in the forebrain at the left midpupillary line, 2 mm posterior to the eye and 2 mm deep from the skin, perpendicular to the skull.^[^ ^18^ ^]^ Each injection contained 1500 PFU of virus in 200 nL. Viral replication was monitored daily using in vivo imaging systems (IVIS) (PerkinElmer, US) with an excitation wavelength of 488 nm for one week. Additionally, mice were anesthetized on days 0, 1, 2, 3, and 7 post‐injection and the whole brain was removed for viral loads test. The whole brain was weighted and then homogenized immediately in 2 mL of cold DMEM, and the virus in the supernatant was tittered via plaque assay on G‐VeroE6 cells. Viral loads in the whole brain of sucking mice were expressed as mean PFU±SD per whole brain for each group.

To create an immunocompromised animal model, female SPF hamsters were treated with 100 mg kg^−1^ of an immunosuppressive agent on Day ‐5, followed by 70 mg kg^−1^ every four days to maintain an immunosuppressed state. For safety evaluations, immunocompromised female hamsters (n = 3 each group) were inoculated with 10^7^ PFU or 10^6^ PFU of VSV_MT_‐S2P, VSV_MT_‐S or VSV‐S in 100 µL of PBS via the IN route. Group details are listed in **Table** **3**. Blood samples were collected on Days ‐5, 0, 7, 14, 21, and 28 d.p.i. for hematology analysis, using WBC count and W‐LCR as indicators. To evaluate the immunogenicity of rVSVs in immunocompromised female hamsters, animals (n = 3 each group) were administered 10^6^ PFU or 10^5^ PFU of VSV_MT_‐S2P or VSV_MT_‐S in 100 µL of PBS via the IN route. Blood samples were collected at 7, 14, 21, and 28 d.p.i. for NAb tests, with BALF harvested at 28 d.p.i. for further testing.

**Table 3:** Groups of cyclophosphamide‐treated hamsters administrated by VSV_MT_‐S2P or VSV_MT_‐S for the evaluations of safety and immunogenicity.

Animal	Inoculum	Routes	Immunization dose	
Safety in CP‐treated hamsters	VSV_MT_‐S2P_BA.5_	IN^a)^	10^6^ PFU	10^7^ PFU
	VSV_MT_‐S2P_BA.1_	IN	10^6^ PFU	10^7^ PFU
	VSV_MT_‐S_BA.5_	IN	10^6^ PFU	10^7^ PFU
	VSV_MT_‐S_BA.1_	IN	10^6^ PFU	10^7^ PFU
	VSV_ΔG_‐S_BA.5_	IN	10^6^ PFU	10^7^ PFU
	VSV_ΔG_‐S_BA.1_	IN	10^6^ PFU	10^7^ PFU
	PBS	IN	NA	
Immunogenicity in CP‐treated hamsters	VSV_MT_‐S2P_BA.5_	IN	10^5^ PFU	10^6^ PFU
	VSV_MT_‐S2P_BA.1_	IN	10^5^ PFU	10^6^ PFU
	VSV_MT_‐S_BA.5_	IN	10^5^ PFU	10^6^ PFU
	VSV_MT_‐S_BA.1_	IN	10^5^ PFU	10^6^ PFU
	PBS	IN	NA	

Female SPF hamsters (n = 3 each group) were initially treated with 100 mg kg^−1^ of cyclophosphamide on Day −5 followed by 70 mg kg^−1^ dose at 4 day intervals to induce immunocompromised states as described in “Methods”. Then the CP‐treated animals were grouped and inoculated with VSV_MT_‐S2P or VSV_MT_‐S viruses in 100 µL PBS or mock treated with PBS via intranasal route.

^a)^ IN: intranasal inoculation.

NA. not applicable; CP: cyclophosphamide.

### Hematology

WBC count and the percentage of large cells relative to white blood cells (W‐LCR) were measured as key indicators of inflammation and immune state. Blood samples (20 µL) from all animals were collected into 1.5 mL tubes containing heparin sodium and analyzed using the pocH‐100iV Diff analyzer (Sysmex, Japan).

### Statistical Analysis

Statistical analyses were performed using SPSS 20.0 (Chicago, USA). The exact tests varied depending on the type of experiment. Differences between two groups were assessed using an unpaired two‐tailed t‐test. Statistical analyses among multiple groups of variance were conducted using a two‐way ANOVA. Significant differences between group means were determined using Tukey's multiple range test. Unless otherwise specified, data are presented as means±SD values. *p*<0.05 was considered statistically significant.

## Conflict of Interest

The authors declare no conflicts of interest.

## Author Contributions

E.Z. and Y.K. contributed equally to this work as co‐first author. T.S., B.Z., E.Z. and Y.K. participated in the conception and design. E.Z., Y.K., W.R., Y.Z., R.L. and L.W. contributed to perform experiments. E.Z. and Y.K. analysis and interpretation of data. E.Z. and Y.K. statistical analysis and drafting of the manuscript. T.S. and B.Z. performed critical revision of the manuscript T.S. and B.Z. final approval of the manuscript.

## Supporting information



Supporting Information

## Data Availability

The data that support the findings of this study are available from the corresponding author upon reasonable request.
