# Supplementary material for: Assessment of Single‐Cycle M‐Protein Mutated Vesicular Stomatitis Virus as a Safe and Immunogenic Mucosal Vaccine Platform for SARS‐CoV‐2 Immunogen Delivery
Source: Adv Sci (Weinh). 2024 Nov 11;11(47):2404197. doi: 10.1002/advs.202404197 (PMC11653642; doi:10.1002/advs.202404197)
Supplement: Supplementary file 1 — Supporting Information [file ADVS-11-2404197-s001.docx]

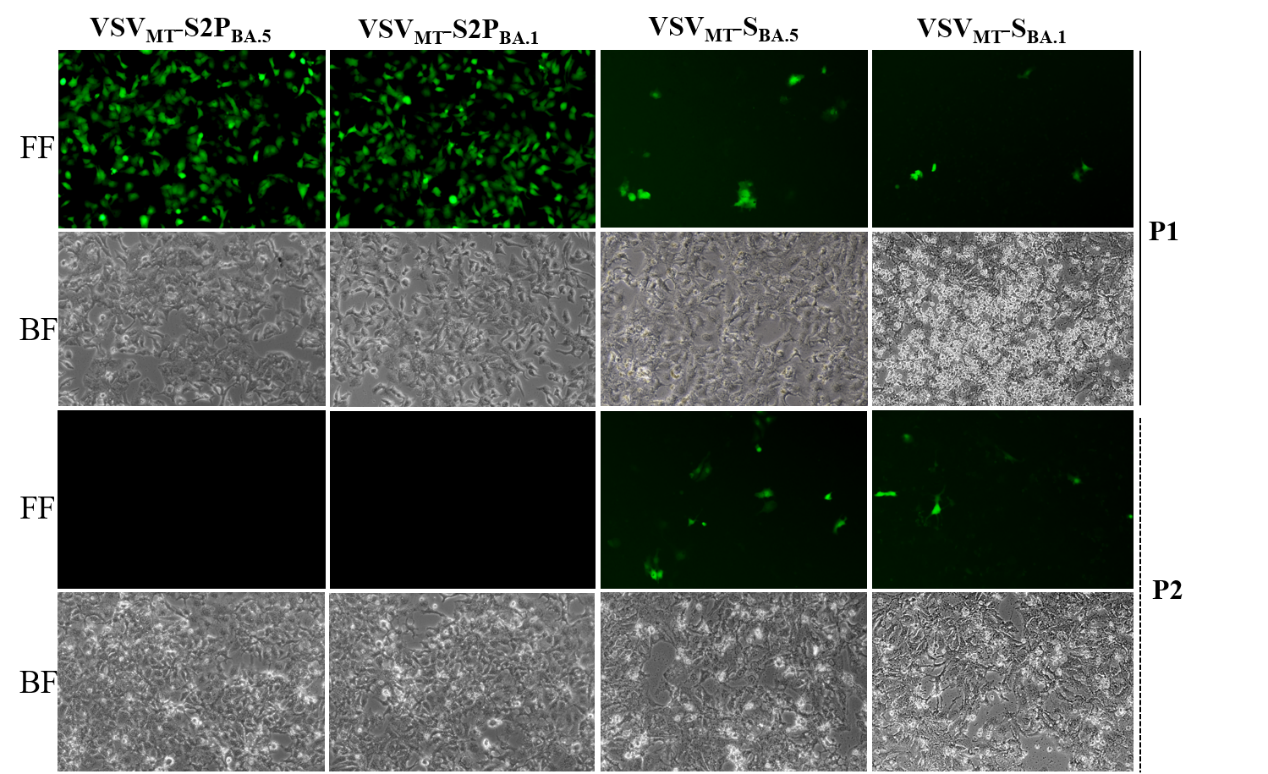


**A**

**Figure S1. Tropism and replication of VSV_MT_-S2P and VSV_MT_-S in Huh-7 cells.** Huh-7 cells were infected with rVSV_MT_-S2P viruses or VSV_MT_-S at MOI of 1. 24 hours post-infection, GFP expression of infected cells was photographed by a fluorescence microscope. The supernatants were then collected and the viral progeny were used to inoculate fresh Huh-7 cells. 24 hours later, the infected cells were also photographed. P1 and P2 represented the first round or second round of infection respectively.


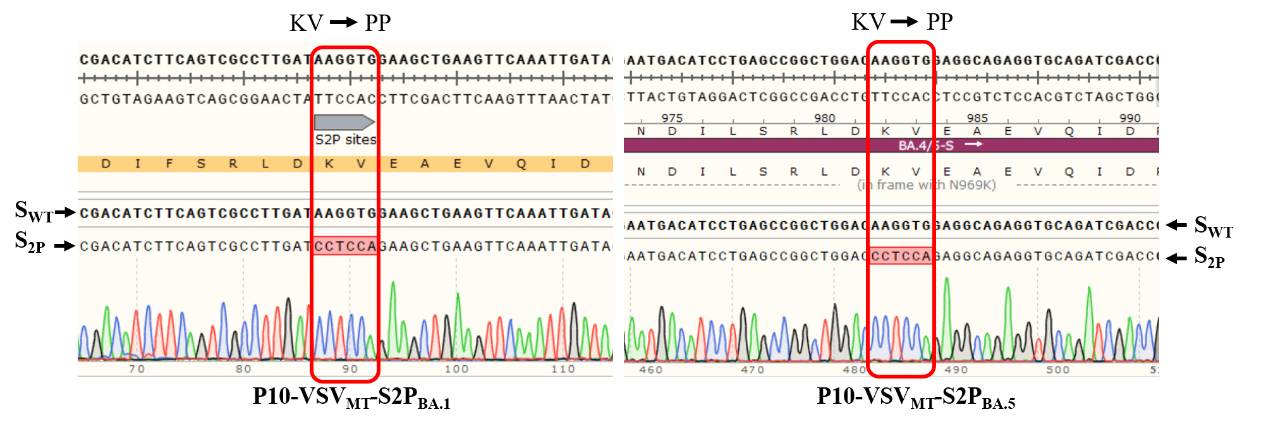


**Figure S2. Genetic stability of the *S2P* gene in VSV_MT_-S2P.** RT-PCR assay was performed to study the genetic stability of VSV_MT_-S2P. The 5th passage for the virus was plaque purified and then serially passaged for up to 10 passages in the BHK21 cells encoding VSV G protein (BHK-G). At least 10 viral plaques were randomly selected from each stock. Viral RNA was extracted from 10th passage of VSV_MT_-S2P and *S2P* gene was amplified after reverse transcription and then sequenced. P10 represented 10th passage. KV: lysine and valine. PP: double proline.

**B**

**C**

**A**

**Figure S3. Viral loads of tissues in Balb/c mice after inoculation with VSV_MT_-S2P via intranasal inoculation.** The VSV N gene CT value was determined by RT-qPCR with turbinalia (A), lung (B) and brain (C) collected at 2, 4, 6 d.p.i.

**A**

| Group | First dose | Subsequent dose |
| --- | --- | --- |
| 140 mg/kg | 140 mg/kg CP at day -5 | 100 mg/kg CP every 4 days |
| 100 mg/kg | 100 mg/kg CP at day -5 | 70 mg/kg CP every 4 days |
| 70 mg/kg | 70 mg/kg CP at day -5 | 50 mg/kg CP every 4 days |
| PBS | PBS at day -5 | PBS every 4 days |

**B**

**C**

**Figure S4**. Cyclophosphamide treatment results in immunosuppression of syrian golden hamsters.

(A) Cyclophosphamide dosing regimens. Groups of animals were injected intraperitoneally with PBS or CP as the concentrations of 140mg/kg, 100mg/kg, and 70mg/kg and schedules to maintain immunosuppression from day 0 to 26 post treatment.

(B) WBC counts per L at days 0~26 post treatment with PBS or various concentrations of CPs as shown in (A). The average and standard error are graphed.

(C) Body weight changes of 140mg/kg CP-treated hamsters which inoculated with VSV_MT_-S2P, VSV_MT_-S or VSV-S via intranasal route. CP was injected intraperitoneally at day -5. At day 0, rVSVs were mock inoculated or inoculated via intranasal route at dose of 5×10^5^ per animal. Animal body weight changes were monitored every day. VSV_ΔG_-S viruses is the replication-competent VSV possessing wild type M ; VSV_MT_-S is that with triple-site mutated M.

| **Concentration**  **(ug/mL)** | **ACE2-Fc** | | | | | | **8G3** | | | | |
| --- | --- | --- | --- | --- | --- | --- | --- | --- | --- | --- | --- |
|  | **IRES-S2P_BA.5_** | **IRES-S_BA.5_** | **IRES-S2P_BA.1_** | **IRES-S_BA.1_** | **BHK21** | **IRES-S2P_BA.5_** | | **IRES-S_BA.5_** | **IRES-S2P_BA.1_** | **IRES-S_BA.1_** | **BHK21** |
| 0.005 | 0.112±0.002 | 0.129±0.004 | 0.093±0.007 | 0.093±0.002 | 0.087±0.016 | 0.066±0.003 | | 0.072±0.005 | 0.081±0.003 | 0.092±0.004 | 0.069±0.001 |
| 0.05 | 0.111±0.002 | 0.144±0.004 | 0.094±0.004 | 0.088±0.001 | 0.090±0.016 | 0.081±0.002 | | 0.077±0.005 | 0.088±0.003 | 0.095±0.002 | 0.068±0.001 |
| 0.5 | 0.133±0.002 | 0.204±0.009 | 0.107±0.002 | 0.101±0.004 | 0.113±0.009 | 0.116±0.004 | | 0.085±0.004 | 0.103±0.002 | 0.100±0.005 | 0.070±0.001 |
| 5 | 0.259±0.016 | 0.474±0.026 | 0.213±0.001 | 0.188±0.009 | 0.114±0.002 | 0.169±0.009 | | 0.105±0.003 | 0.340±0.020 | 0.313±0.014 | 0.067±0.005 |
| 50 | 2.489±0.070 | 2.815±0.212 | 1.539±0.029 | 1.394±0.180 | 0.124±0.015 | 0.198±0.030 | | 0.164±0.014 | 1.254±0.133 | 1.313±0.064 | 0.065±0.006 |
| 100 | 3.055±0.137 | 3.384±0.060 | 3.027±0.087 | 2.533±0.185 | 0.183±0.069 | 0.744±0.081 | | 0,746±0.052 | 2.521±0.134 | 2.489±0.062 | 0.070±0.009 |
| 200 | 3.295±0.030 | 3.404±0.038 | 3.482±0.071 | 3.136±0.037 | 0.067±0.001 | 0.779±0.023 | | 0.772±0.042 | 2.565±0.175 | 2.609±0.173 | 0.070±0.009 |
| 400 | 3.350±0.004 | 3.444±0.020 | 3.530±0.038 | 3.168±0.107 | 0.068±0.002 | 0.806±0.097 | | 0.877±0.037 | 2.766±0.108 | 2.708±0.209 | 0.082±0.004 |

**Table S1.** Affinity activity between SARS-CoV-2 S2P and hACE2-Fc or 8G3 antibody.

The interaction binding activity of S_∆21_2P or full-length S proteins with human ACE2 and BA.1 specific antibody 8G3 were detected with cell-based ELISA. The mutant genes of BA.1, BA.5 strains were initially transfected into BHK21 cells, with the parental S genes as the controls. After 24 h, the cells expressing S2P or S protein were incubated with different concentration of hACE2-Fc or 8G3 antibody respectively. The interaction binding activity were detected with cell-based ELISA.

| **Tissue** | **Dpi** | **VSV_MT_-S2P_BA.1_** | | **VSV_MT_-S_BA.1_** | | **VSV_MT_-S2P_BA.5_** | | **VSV_MT_-S_BA.5_** | | **PBS** |
| --- | --- | --- | --- | --- | --- | --- | --- | --- | --- | --- |
|  |  | **10^7^** | **10^6^** | **10^7^** | **10^6^** | **10^7^** | **10^6^** | **10^7^** | **10^6^** | **NA** |
| **Turbinate** | **2** | 34.46±0.26 | 34.26±0.56 | 34.42±0.71 | 34.34±0.27 | 34.93±0.10 | 35.19±0.50 | 34.76±0.59 | 34.89±0.32 | 34.59±0.50 |
|  | **4** | 34.53±0.17 | 34.54±0.09 | 34.39±0.22 | 35.15±0.87 | 34.58±0.25 | 35.04±0.37 | 34.64±0.51 | 34.98±0.19 | 34.93±0.51 |
|  | **6** | 34.55±0.57 | 34.06±0.36 | 34.09±0.04 | 34.26±0.47 | 35.45±0.32 | 35.30±0.57 | 35.26±0.55 | 34.87±0.20 | 34.93±0.41 |
| **Lung** | **2** | 34.49±0.43 | 34.33±0.03 | 34.22±0.22 | 33.87±0.13 | 34.58±0.28 | 34.03±0.78 | 34.57±0.41 | 34.94±0.46 | 34.38±0.49 |
|  | **4** | 34.35±0.09 | 34.39±0.17 | 34.17±0.35 | 34.37±0.28 | 34.91±0.43 | 34.64±0.64 | 35.21±0.33 | 35.03±0.21 | 35.19±0.49 |
|  | **6** | 33.91±0.29 | 34.18±0.38 | 34.15±0.16 | 33.58±0.09 | 34.47±0.36 | 34.55±0.31 | 34.95±0.11 | 34.74±0.55 | 34.58±0.46 |
| **Brain** | **2** | 34.64±0.16 | 34.88±0.12 | 34.38±0.16 | 34.55±0.08 | 34.94±0.15 | 34.61±0.22 | 34.75±0.43 | 34.54±0.33 | 34.39±0.38 |
|  | **4** | 34.83±0.04 | 34.71±0.16 | 34.73±0.25 | 34.66±0.12 | 34.43±0.48 | 34.09±0.64 | 34.68±0.14 | 34.71±0.15 | 34.67±0.05 |
|  | **6** | 34.49±0.31 | 34.58±0.49 | 34.51±0.35 | 34.71±0.25 | 34.82±0.19 | 34.93±0.33 | 34.76±0.27 | 34.44±0.19 | 34.53±0.1 |

**Table S2.** The CT value for turbinate, lung and brain in LVG Hamster

NA, no applicable. The VSV N gene CT value for turbinalia, lung and brain in LVG hamster collected at 2, 4, 6 d.p.i was determined by RT-qPCR.

| **VSV_MT_-** | **Dose** | **7 dpi** | | | **14 dpi** | | | **21 dpi** | | | | **28 dpi** | | | |
| --- | --- | --- | --- | --- | --- | --- | --- | --- | --- | --- | --- | --- | --- | --- | --- |
|  |  | **IM** | **IN** | **Mock** | **IM** | **IN** | **Mock** | **IM** | **IN** | **Mock** | **IM** | | **IN** | **Mock** |  |
| **S_BA.1_** | 10^6^ | 427±185 | 1707±739 | - | 533±185 | 1707±739 | - | 533±185 | 2133±739 | - | 853±369 | | 4266±1478 | - |  |
|  | 10^5^ | 213±92 | 853±369 | - | 213±92 | 853±369 | - | 373±244 | 1707±739 | - | 747±489 | | 3413±1478 | - |  |
| **S2P_BA.1_** | 10^6^ | 267±92 | 57±40 | - | 533±185 | 67±23 | - | 640 | 213±92 | - | 1707±739 | | 373±244 | - |  |
|  | 10^5^ | 120±69 | 7±6 | - | 160 | 30±17 | - | 267±92 | 67±23 | - | 267±92 | | 80 | - |  |
| **S_BA.5_** | 10^6^ | 853±369 | 2133±739 | - | 1067±369 | 3413±1478 | - | 1707±739 | 6826±2956 | - | 1707±739 | | 6826±2956 | - |  |
|  | 10^5^ | 320 | 1493±978 | - | 427±185 | 1493±978 | - | 533±185 | 2133±739 | - | 533±185 | | 2560 | - |  |
| **S2P_BA.5_** | 10^6^ | 120±69 | 7±6 | - | 160 | 20 | - | 960±554 | 20 | - | 960±554 | | 67±23 | - |  |
|  | 10^5^ | 40 | 7±6 | - | 160 | 7±6 | - | 267±92 | 7±6 | - | 267±92 | | 7±6 | - |  |

**Table S3.** Serum neutralizing antibody titers in LVG hamster inoculated with VSV_MT_-S2P

IN. intranasal inoculation

IM. intramuscular injection.

-. undetable. Nab titers were expressed as the reciprocal of the highest dilution of antibody giving a 100% inhibition of cytopathic effect.

**Table S4.** The VSV N gene CT value for turbinate, lung and brain in Balb/c mice

| **Tissue** | **Dpi** | **VSV_MT_-S2P_BA.1_** | | | **VSV_MT_-S2P_BA.5_** | | | **PBS** |
| --- | --- | --- | --- | --- | --- | --- | --- | --- |
|  |  | **2.5×10^6^** | | **2.5×10^5^** | **2.5×10^6^** | | **2.5×10^5^** | NA |
| **Turbinate** | 2 | 34.62±0.41 | 34.50±0.34 | | 34.11±0.13 | 33.93±0.16 | | 34.54±0.17 |
|  | 4 | 34.25±0.27 | 34.44±0.29 | | 34.77±0.28 | 33.96±0.29 | | 34.94±0.61 |
|  | 6 | 34.36±0.30 | 34.33±0.44 | | 34.63±0.44 | 34.53±0.18 | | 34.57±0.40 |
| **Lung** | 2 | 34.60±0.40 | 34.70±0.54 | | 34.66±0.27 | 34.94±0.23 | | 35.09±0.53 |
|  | 4 | 34.63±0.45 | 35.10±0.45 | | 34.64±0.46 | 35.21±0.23 | | 34.54±0.15 |
|  | 6 | 34.55±0.42 | 34.75±0.70 | | 35.23±0.44 | 35.05±0.23 | | 34.54±0.42 |
| **brain** | 2 | 34.60±0.40 | 34.70±0.37 | | 34.64±0.49 | 34.83±0.10 | | 34.79±0.21 |
|  | 4 | 34.96±0.20 | 34.81±0.30 | | 34.98±0.34 | 35.07±0.52 | | 35.09±0.53 |
|  | 6 | 35.04±0.31 | 34.60±0.52 | | 35.17±0.39 | 35.36±0.13 | | 34.63±0.25 |

NA, no applicable. The VSV N gene CT value for turbinalia, lung and brain in Balb/c mice collected at 2, 4, 6 d.p.i was determined by RT-qPCR.

**Table S5.** Serum neutralizing antibody titers in Balb/c mice inoculated with VSV_MT_-S2P

IN. intranasal inoculation

IM. intramuscular injection.

-. undetable. Nab titers were expressed as the reciprocal of the highest dilution of antibody giving a 100% inhibition of cytopathic effect.

| **VSV_MT_-S2P** | **Dose** | **7 dpi** | | | **14 dpi** | | | **21 dpi** | | | **28 dpi** | | |
| --- | --- | --- | --- | --- | --- | --- | --- | --- | --- | --- | --- | --- | --- |
|  |  | **IM** | **IN** | **Mock** | **IM** | **IN** | **Mock** | **IM** | **IN** | **Mock** | **IM** | **IN** | **Mock** |
| **BA.1** | 2.5×10^5^ | 80±80 | 23±15 | - | 173±140 | 53±23 | - | 267±92 | 133±46 | - | 427±185 | 213±92 | - |
|  | 2.5×10^4^ | 17±6 | 8±3 | - | 160 | 40 | - | 267±92 | 67±23 | - | 320±277 | 187±122 | - |
| **BA.5** | 2.5×10^5^ | 80±69 | 3±6 | - | 187±122 | 33±40 | - | 480±277 | 43±35 | - | 747±489 | 60±35 | - |
|  | 2.5×10^4^ | 10 | 3±6 | - | 123±171 | 0 |  | 253±335 | 0 | - | 187±122 | 0 | - |

**Table S6.** T cell-mediated immune response in Balb/c mice inoculated with VSV_MT_-S2P

| **VSV_MT_-S2P** | **Dose** | **S1 pool peptide** | | | | | **S2 pool peptide** | | |
| --- | --- | --- | --- | --- | --- | --- | --- | --- | --- |
|  |  | **IM** | **IN** | | **Mock** | | **IM** | **IN** | **Mock** |
| **BA.1** | 2.5×10^5^ | 156.7±2.48 | | 57.6±1.85 | | 4.3±2.62 | 38.8±1.77 | 35.4±4.13 | 4±2.16 |
|  | 2.5×10^4^ | 82.9±2.10 | | 15.2±1.55 | |  | 37.4±2.33 | 14.1±1.52 |  |
| **BA.5** | 2.5×10^5^ | 190.0±5.03 | | 36.4±2.76 | | 6.7±2.87 | 77.2±2.09 | 11.8±1.32 | 3±2.16 |
|  | 2.5×10^4^ | 63.6±4.55 | | 8.7±1.49 | |  | 56.1±2.85 | 3.6±0.28 |  |

IN. intranasal inoculation

IM. intramuscular injection. IFN-γ levels were detected using ELISPOTS assay. S1 and S2 pool peptide represented a panel of epitopes in S1 or S2 of SARS-CoV2 S protein .

**Table S7.** Serum neutralizing antibody titers in immunocompromised LVG hamster inoculated with VSV_MT_-S2P

| **VSV_MT_-** | **Dose** | **7 dpi** | | **14 dpi** | | **21 dpi** | | **28 dpi** | |
| --- | --- | --- | --- | --- | --- | --- | --- | --- | --- |
|  |  | **IN** | **Mock** | **IN** | **Mock** | **IN** | **Mock** | **IN** | **Mock** |
| **S2P_BA.1_** | 10^6^ | 0 | - | 7±6 | - | 17±6 | - | 17±6 | - |
|  | 10^5^ | 0 | - | 0 | - | 7±6 | - | 7±6 | - |
| **S_BA.1_** | 10^6^ | 7±6 | - | 53±23 | - | 187±122 | - | 240±138 | - |
|  | 10^5^ | 0 | - | 8±3 | - | 17±6 | - | 60±35 | - |
| **S2P_BA.5_** | 10^6^ | 0 | - | 10 | - | 17±6 | - | 30±17 | - |
|  | 10^5^ | 0 | - | 3±6 | - | 13±6 | - | 13±6 | - |
| **S_BA.5_** | 10^6^ | 10±10 | - | 67±23 | - | 267±92 | - | 267±92 | - |
|  | 10^5^ | 0 | - | 10 | - | 23±15 | - | 60±35 | - |

IN. intranasal inoculation

-. Undetected. Nab titers were expressed as the reciprocal of the highest dilution of antibody giving a 100% inhibition of cytopathic effect.
